# A test of indirect grounding of abstract concepts using multimodal distributional semantics

**DOI:** 10.3389/fpsyg.2022.906181

**Published:** 2022-10-04

**Authors:** Akira Utsumi

**Affiliations:** Department of Informatics, Artificial Intelligence Exploration Research Center, The University of Electro-Communications, Tokyo, Japan

**Keywords:** abstract concepts, indirect grounding, embodied cognition, multimodal distributional semantic model, conceptual representation, symbol grounding problem

## Abstract

How are abstract concepts grounded in perceptual experiences for shaping human conceptual knowledge? Recent studies on abstract concepts emphasizing the role of language have argued that abstract concepts are grounded indirectly in perceptual experiences and language (or words) functions as a bridge between abstract concepts and perceptual experiences. However, this “indirect grounding” view remains largely speculative and has hardly been supported directly by empirical evidence. In this paper, therefore, we test the indirect grounding view by means of multimodal distributional semantics, in which the meaning of a word (i.e., a concept) is represented as the combination of textual and visual vectors. The newly devised multimodal distributional semantic model incorporates the indirect grounding view by computing the visual vector of an abstract word through the visual vectors of concrete words semantically related to that abstract word. An evaluation experiment is conducted in which conceptual representation is predicted from multimodal vectors using a multilayer feed-forward neural network. The analysis of prediction performance demonstrates that the indirect grounding model achieves significantly better performance in predicting human conceptual representation of abstract words than other models that mimic competing views on abstract concepts, especially than the direct grounding model in which the visual vectors of abstract words are computed directly from the images of abstract concepts. This result lends some plausibility to the indirect grounding view as a cognitive mechanism of grounding abstract concepts.

## 1. Introduction

### 1.1. Abstract concepts and embodied cognition

Since Harnad (1990) pointed out the symbol grounding problem, embodied approaches to cognition have emerged as promising solutions of how symbols (or words) acquire their meanings. Embodied cognition theories argue that concepts or word meanings are grounded in our perceptual or sensorimotor experiences. For example, Barsalou's (1999) theory of perceptual symbol systems states that concepts (and word meanings as well) are inherently modal, perceptual symbols grounded in the real world. Perceptual symbols refer to neural representations, or activation patterns of sensorimotor regions of the brain elicited during direct perceptual experiences (e.g., seeing, touching, and playing with dogs for the concept of dog). Once these representations are encoded in the brain by repeated experiencing, they can be reactivated, that is, experiences are mentally simulated when words are encountered even in the absence of direct experience. Embodied cognition theories have been empirically supported by a considerable number of studies (e.g., Glenberg and Kaschak, 2002; Kaschak et al., 2005; Pecher and Zwaan, 2005; Barsalou, 2008; Pulvermüller, 2013; Scorolli, 2014; Barsalou, 2016; Coello and Fisher, 2016; Fisher and Coello, 2016).

However, abstract concepts pose a serious challenge to the embodied theory of cognition. Because abstract concepts such as *love* and *justice* do not have clearly perceivable referents, it is difficult to see how representations grounded in perceptual experiences can capture the content of abstract concepts. The empirical studies on embodied cognition have previously focused primarily on concrete concepts, such as *dog* and *kick*, which directly refer to perceivable objects or physical motions. Recently, however, the focus of concepts research has recently shifted from the embodied nature of concrete concepts to the complex nature of abstract concepts (Bolognesi and Steen, 2018; Borghi et al., 2018).

Some embodied theories claim a general mechanism of grounding common to both concrete and abstract concepts. Barsalou (1999, 2003) advocates that abstract concepts are represented in the same perceptual symbol systems. Perceptual symbols for abstract concepts are acquired from sensorimotor and introspective experiences in specific situations and abstract words elicit mental simulations of those situations. For example, people visualize and emotionalize two people kissing when seeing the word “love” and the court when seeing the word “justice.” A recent view of situated simulation is more radical; Barsalou et al. (2018) proposed that the distinction between concrete and abstract concepts is no longer useful and should be abandoned because all concepts can be explained within the situated simulation view. The situated simulation view is supported by a number of empirical studies. Barsalou and Wiemer-Hastings (2005) and Wiemer-Hastings and Xu (2005) found using a property generation task that, when participants generated properties for abstract concepts, they were likely to describe social and introspective aspects of the situations, whereas for concrete concepts they tended to describe properties of entities in the situations. McRae et al. (2018) demonstrated that pictures of specific situations facilitated lexical decisions to abstract words relevant to the picture primes, and conversely abstract words also facilitated processing of pictures depicting the relevant situations. These empirical findings may suggest that the situated simulation view is plausible and at least partially resolves the problem of how abstract concepts are grounded in our perceptual or sensorimotor experiences.

The situated simulation view, however, is not essentially sufficient to explain abstract concepts. Imagine that you have to explain abstract concepts using only visual images or videos without language. For example, to explain what is love, people may show a picture of two people kissing and hugging, a picture of wedding ceremony, and/or a picture of specific dating situations. These pictures can convey some conceptual knowledge about love, and more elaborate visual images such as films can convey greater knowledge. We feel nevertheless that only seeing them lacks something to fully understand the concept of love. This difficulty becomes more serious when more abstract concepts (e.g., *justice* and *democracy*) have to be explained; they are more difficult to explain using only visual images. This simple thought experiment suggests that situated simulation is somewhat limited as a thorough theory of abstract concepts. Another limitation is that the situated simulation view seems not to provide a clear explanation of how abstract concepts are linked to the relevant situations in acquiring those concepts. It is much less likely that people think of abstract concepts (e.g., *democracy*), or see or hear abstract words that refer to those concepts, at the same time as experiencing the situations associated with those concepts (e.g., *casting a vote in a polling station*), in contrast to concrete concepts (e.g., *dog*), which are often mentioned verbally in the situations including their referents. through a video-corpus analysis of mother–infant interaction that mothers used abstract words less often in the presence of their referent events than they used concrete words in the presence of their referent objects.

These limitations of the situated simulation view can be largely overcome by taking into account language not only as a source of conceptual knowledge but also as an effective means of grounding abstract concepts in the real world. It has been widely accepted that language is much more important for representing abstract concepts (e.g., Borghi et al., 2017; Dove, 2018). Neuroimaging studies have demonstrated that processing of abstract concepts elicits greater activation of the left-dominant Perisylvian language network (including the left inferior frontal gyrus and the left superior temporal cortex) as compared to processing of concrete concepts (e.g., Binder et al., 2009; Wang et al., 2010). Recent embodied theories of abstract concepts have therefore emphasized the role of language in forming and processing abstract concepts or words (Borghi et al., 2017; Bolognesi and Steen, 2018). One of the important questions to be addressed by these theories is how language and embodied experience contribute to shaping our conceptual knowledge of abstract concepts and meaning representation of abstract words (e.g., Bolognesi and Steen, 2018). This question is what we address in this paper.

### 1.2. Hybrid theory integrating symbolic and embodied cognition

The dual coding theory (Paivio, 1971, 1986) is an early influential theory that integrates symbolic and embodied cognition. The main claim of this theory is that concepts are represented in two separate systems, that is, a verbal system for linguistic information and a visual system for mentally visual images. Furthermore, the dual coding theory argues that concrete words activate both the verbal and visual systems, but abstract words activate only the verbal system. This argument is consistent with the concreteness effect, whereby concrete words have processing and mnemonic advantages over abstract words. By contrast, it implies that abstract words are represented primarily by linguistic information, and it is not clear whether and how the visual system contributes to the representation of abstract words. To explain the concreteness effect, Schwanenflugel et al. (1988) and Schwanenflugel (1991) also proposed the context availability theory. According to this theory, concrete words are strongly associated with a few contexts, whereas abstract words are weakly associated with many contexts. Therefore, the context availability theory can explain the concreteness effect because abstract words require more effort to activate their contexts. Although contexts in this theory can be both linguistic or embodied, this theory is not devoted to the differences and relations between linguistic and embodied contexts or representations.

Recent hybrid theories, which are collectively referred to as “multiple representation theories” (Borghi et al., 2017), are more committed to how language processing interacts with embodied cognition. The multiple representation theory proposed first is Language And Situated Simulation (LASS) theory (Barsalou et al., 2008). The LASS theory focuses on the temporal interplay between language processing and situated simulation during conceptual processing. According to the LASS theory, when a word is perceived, both linguistic and simulation systems become active initially, but the linguistic system becomes engaged immediately to categorize the word. For the tasks requiring only shallow comprehension (e.g., lexical decision task), language processing would suffice. When deeper conceptual processing (e.g., property generation task) is required, the simulation system is activated later after the activation of the linguistic system peaks. However, the LASS theory is not aimed at explaining how abstract concepts are represented. It claims that both concrete and abstract concepts activate a mixture of linguistic and embodied information, and which information is dominant is determined depending on the task^1^, not the concept. The situated simulation view still holds in this framework, and thus the LASS theory also suffers from the limitations of the situated simulation view described in section 1.1.

A more influential theory for the multiple representation views of abstract concepts is the “Words As social Tools” (WAT) theory (Borghi et al., 2013; Borghi and Binkofski, 2014; Borghi et al., 2019). The WAT theory claims that abstract concepts depend more on language than concrete concepts, but the role of language is not limited to word association. It emphasizes the importance of language (or words) as tools to perform social actions, and argues that the situated simulation (i.e., re-enactment) of social experience through language is necessary for representing and acquiring abstract concepts. The WAT theory is supported by a number of empirical findings on language acquisition and brain organization (see, Borghi et al., 2017, 2019), but it does not clearly explain the mechanism of how language shapes the meaning representation of abstract concepts. Words not only are tools for direct social experiencing of language-related actions and events, but also function as a bridge to direct perceptual and sensorimotor experience, which is the main tenet of the indirect grounding view described in the next section.

### 1.3. Indirect grounding view

The hybrid views mentioned above assume that language provides a separate source of conceptual knowledge independent of embodied experience or the use of language is itself a constituent of embodied experience in which abstract concepts are grounded. Unlike the hybrid views, some recent studies have been devoted specifically to how language is used to relate abstract concepts to embodied experience.

The symbol interdependency hypothesis proposed by Louwerse (2011, 2018) argues for the role of language as a shortcut to the perceptual or embodied system. According to the symbol interdependency hypothesis, language comprehension is symbolic through interdependencies of amodal linguistic symbols, while it is indirectly embodied through the references linguistic symbols make to perceptual representations. Hence, “language has evolved to become a communicative short-cut for language users and encodes relations in the world, including embodied relations (Louwerse, 2011, p. 279).” language provides an important means of extending our cognitive capabilities and encoding abstract concepts by enabling access to an embodied representational system that exists independently of language. Their “division of labor” approach assumes two layers of conceptual processing; a perceptual layer that associates basic, concrete concepts with perceptual features and a relational (i.e., linguistic) layer that grounds more complex and abstract concepts in relation to basic concepts. The relational layer can be formed by the interdependency of linguistic symbols obtained through distributional learning. Lupyan and Lewis's (2019) “words-as-cues” view is consistent with these views; they argue that language provides a cue to meaning that can augment semantic knowledge derived from perceptual experiences or construct semantic knowledge.

In fact, the same line of thought has been suggested earlier in the context of word learning or language acquisition. an abstract word inherits some meaning from the concrete words to which it is related. In their words, “the grounded meaning propagates up through the syntactic links of the co-occurrence meaning network, from the simplest early words to the most abstract (Howell et al., 2005, p. 260).” along the same line. In the early stage of lexical acquisition, the meaning of concrete words is acquired directly from perceptual information via word-to-world pairing. In the later stage, the meaning of hard words, which is not easily accessible through perception, is acquired by a structure-to-world mapping procedure that combines linguistic observations with co-occurring perceptual experience.

The basic idea underlying all these views is that abstract concepts or the meaning of abstract words are grounded in sensorimotor or perceptual experiences, but the grounding is indirect, rather than direct in the case of concrete concepts. Language not only provides a means to understand and represent abstract concepts (and the meaning of abstract words as well) through statistical regularities in linguistic surface structure, but also functions as *a mediator* between abstract concepts and perceptual experiences for a deeper understanding of abstract concepts in the absence of direct experiences with the words referring to the abstract concepts. For example, people can understand the concept of love by associating the word “love” with “kiss” (and many other relevant mediator words) via linguistic interdependency and mentally simulating the situation of kissing, even though they have never encountered the word “love” directly in the situation of kissing. In this paper, we collectively refer to these views as *indirect grounding views*.

Although the indirect grounding view may be able to provide a promising solution for the symbol grounding problem of abstract concepts, it remains largely speculative and has hardly been supported directly by empirical evidence. To empirically justify the symbol interdependency hypothesis, Louwerse and Jeuniaux (2010) demonstrated that the symbolic factor (i.e., frequency of word pairs) predicted error rates and response time in both semantic and iconicity judgments, whereas the embodied factor (i.e., iconic configuration) predicted error rates and response time in iconicity judgment. using both concrete and abstract words as stimuli and found that, for both concrete and abstract words, the symbolic factor dominated in semantic judgment and embodied factor dominated in iconicity judgment. Although these findings support the general claim that language comprehension is both embodied and symbolic, which can be predicted by the indirect grounding view and even by some of the hybrid views, they do not provide direct evidence for indirect grounding of abstract words.

Recently, Günther et al. (2020) provided more direct evidence for the indirect grounding view using an experimental paradigm (e.g., Zwaan and Yaxley, 2003) in which target words are faster to process when their perceptually embodied meaning (e.g., spatial location) is congruent with perceptual experiences that participants have in the experiment. They applied this paradigm to new concepts for which participants had no direct perceptual experience, but which they learned from language alone referring to vertical (i.e., up or down) concepts. The result was that, after learning new concepts via language, participants were faster at responding to sentences describing those concepts when their implied vertical position matched the direction of their hand movement for responding. This finding indicates that novel (unknown) concepts, even though not grounded directly, can be grounded indirectly by establishing a connection with directly grounded concepts via language network. In this paper, we test further the validity of the indirect grounding view for existing abstract concepts, in particular the role of language in the grounded representation of abstract concepts, using another methodology, that is, by means of multimodal distributional semantics described next.

### 1.4. Multimodal distributional semantics

Distributional semantics is an effective computational approach to constructing word meaning representations (i.e., word vectors) from the distributional statistics of words in large collections of text (Turney and Pantel, 2010; Lenci, 2018; Pilehvar and Camacho-Collados, 2020). Distributional semantics has been widely used in natural language processing (NLP) as meaning representations for neural networks or deep learning (Goldberg, 2017) and in cognitive science as a cognitive modeling method (Jones et al., 2015; Kumar, 2021). In cognitive research on concepts, in particular on embodied vs. symbolic processing, distributional semantics is regarded as a de facto standard language model (de Vega et al., 2008; Bolognesi and Steen, 2018).

Distributional semantics has been criticized as psychologically implausible because it is based only on linguistic (i.e., symbolic) information and thus suffers from the symbol grounding problem (de Vega et al., 2008; Baroni, 2016). Although it is controversial whether distributional semantics cannot essentially capture human semantic or conceptual knowledge, it is undoubtedly unable to represent the meaning of some kinds of words, in particular concrete words, just as they are represented in human semantic memory.

An earlier approach to this problem is to integrate feature-based information, which is often produced by humans in property generation tasks, with distributional semantics (Andrews et al., 2009; Johns and Jones, 2012; Silberer and Lapata, 2012; Hill and Korhonen, 2014). For example, Andrews et al. (2009) used perceptual features collected as featural norm to ground a language-based topic model on perceptual experience, and demonstrated that the integrated model outperformed the language-based topic model. However, the grounding ability of these feature-integrated models is not sufficient for modeling embodied cognition. Perceptual features produced by humans are limited to what can be conveyed verbally, and they are often only salient and distinctive. Hence, implicit perceptual features characterizing a concept cannot be taken into account in the models (Bruni et al., 2014). Additionally, the number of concepts (or words) used in experiments with human-generated properties is relatively small.

A more promising and common approach is to directly integrate non-verbal information with (text-based) distributional semantics. Multimodal distributional semantics has been proposed for this purpose (for a review, see Baroni, 2016). In multimodal distributional semantics, linguistic or textual information is integrated with perceptual information computed directly from non-linguistic inputs such as visual (Bruni et al., 2014; Kiela et al., 2014; Silberer et al., 2017), auditory (Kiela and Clark, 2015), or olfactory (Kiela et al., 2015) ones. Furthermore, another type of approach has been proposed which utilizes visual information without directly computing visual vectors from images. Bolognesi's (2016, 2017) Flickr^®^ Distributional Tagspace uses the co-occurrence statistics between user-generated tags that appear in the same image to generate word vectors. In this paper, we use multimodal distributional semantics to model the hybrid view of conceptual representation.

Multimodal distributional semantic models generally compute and utilize perceptual vectors in the same way for all words; they do not take account of the difference between concrete and abstract words in terms of how concepts are grounded in perceptual information, which is claimed by the indirect grounding view. Furthermore, it has been empirically demonstrated that a simple addition of perceptual information is beneficial only for concrete concepts (Bruni et al., 2014; Kiela et al., 2014). Therefore, in section 2, we devise a new multimodal distributional semantic model for indirect grounding by incorporating the indirect grounding view into an algorithm for constructing multimodal word vectors. In the devised model, the perceptual vector of an abstract word is computed from the perceptual representations of concrete words that are semantically related to (or associated with) that abstract word.

It must be noted that technically word vectors in (both unimodal and multimodal) distributional semantics are regarded as representing the meaning (or semantics) of words. In this paper, however, we consider the meaning of words and concepts (or the conceptual knowledge) as interchangeable, as is usually assumed in the cognitive science literature (e.g., Vigliocco and Vinson, 2007; Jackendoff, 2019). Although we do not intend to argue that concepts and word meanings are the same, it is much difficult or impossible to distinguish between concepts and word meanings in most of the cases; empirical studies on embodied cognition have used words for the tasks on conceptual representation in human adults (Borghi et al., 2017). In what follows, therefore, we assume that distributional word vectors also represent the concept referred to by a word, and that multimodal distributional semantics can be applied to modeling embodied conceptual processing.

### 1.5. Aim of this study

The aim of the present study is to test the indirect grounding view of abstract concepts by using computational modeling based on multimodal distributional semantics. For this purpose, we compare the indirect grounding view and other competing views mentioned above, the basic tenets of which are summarized in Table 1. By examining which of these views can predict the performance difference among distributional semantic models that mimic these views (and other baseline models), we attempt to test the validity of the indirect grounding view. The performance of distributional semantic models is evaluated in terms of the degree to which human conceptual representation can be predicted by the models, using Utsumi's (2020) experimental framework for analyzing and evaluating distributional semantic vectors. In the evaluation experiment, we focus on visual images as a source of perceptual (i.e., non-verbal) information, as used in many other studies on multimodal distributional semantics.

**Table 1:** Summary of conceptual representation theories.

**Theory**	**Basic tenets**	**Processing difference between abstract and concrete concepts**
Situated simulation view (e.g., Barsalou, 1999; Barsalou et al., 2018)	• Concepts are grounded in perceptual experiences via mental simulation. • Language information is not necessary for understanding concepts.	No
Dual coding theory citepPaivio71,Paivio86	• Concrete concepts are both linguistic and grounded in perceptual experiences. • Abstract concepts are only linguistic.	Yes
Hybrid view (e.g., Barsalou et al., 2008; Borghi et al., 2019)	• Concepts are both linguistic and grounded in perceptual experiences. • The mechanism of grounding does not differ between concrete and abstract concepts; both concepts are grounded directly.	No
Indirect grounding view (e.g., Howell et al., 2005; Louwerse, 2011)	• Concepts are both linguistic and grounded in perceptual experiences. • Abstract concepts are grounded indirectly via language, whereas concrete concepts are grounded directly.	Yes

In the rest of this paper, after describing in detail a new multimodal semantic model for simulating indirect grounding in section 2, we explain the method of our evaluation experiment in section 3. We then report the results of the evaluation experiment in section 4 and discuss the implications and limitations of the findings in section 5.

## 2. Distributional semantic model for indirect grounding

**Figure 1:**
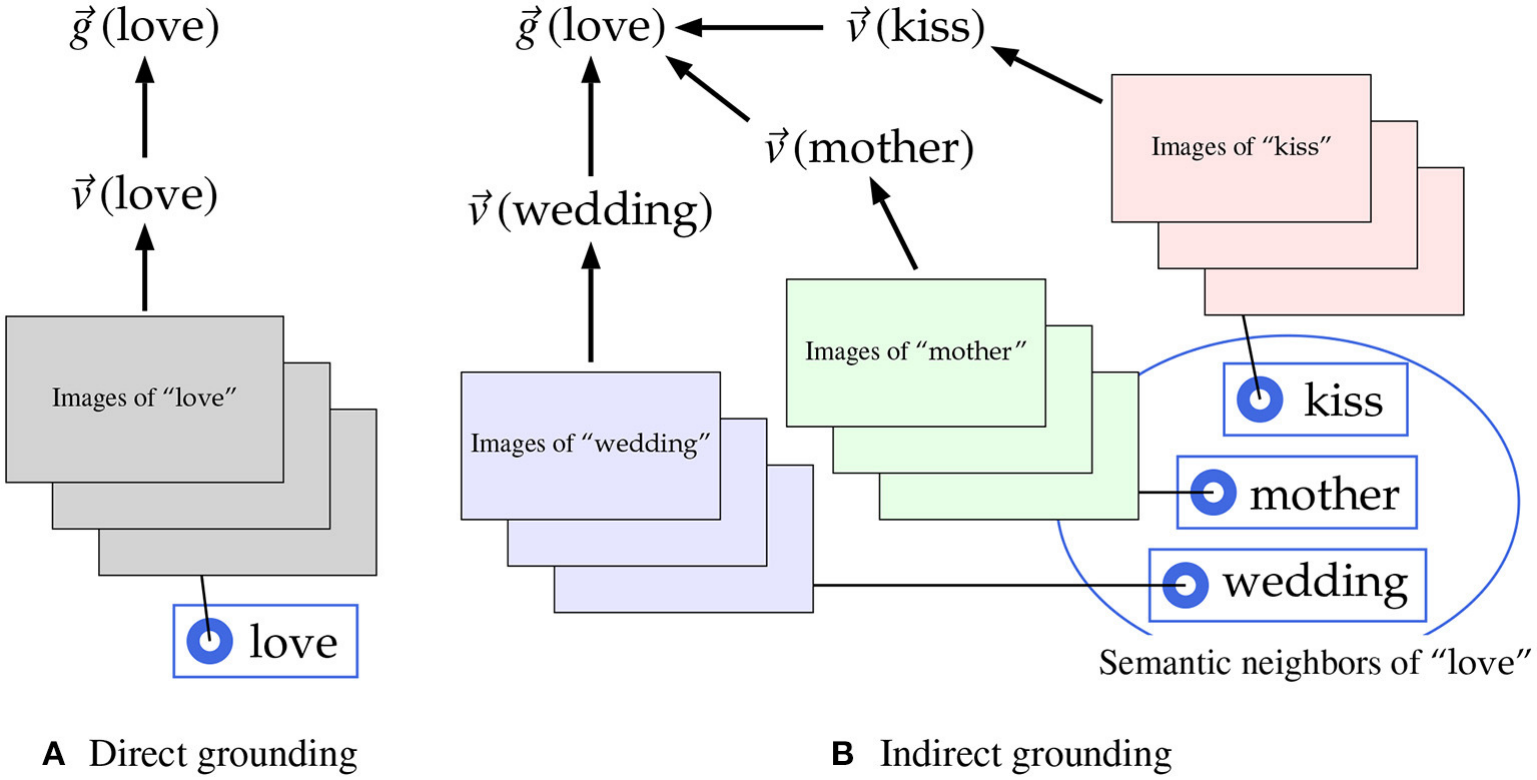
Two methods to compute the visual vector in the multimodal distributional semantic model for indirect grounding. **(A)** Direct grounding. **(B)** Indirect grounding.

We devise a new model to incorporate the indirect grounding view into multimodal distributional semantics. According to the indirect grounding view arguing that grounding of abstract concepts is mediated by language, different methods are used for computing visually grounded vectors depending on whether words are concrete or abstract. For a concrete word, its visually grounded vector is computed directly from the visual images tagged with that word, as shown in Figure 1A. For example, starting from the blind and not knowledgeable assumption that “love” is a concrete word, the visually grounded vector of “love” is computed directly from the images tagged with “love” using deep neural networks (DNNs). In this case, the visually grounded vector $\vec{g}\left(\text{love}\right)$ is identical to the directly computed visual vector $\vec{v}\left(\text{love}\right)$, as generally assumed by multimodal distributional semantic models that take no account of the difference between concrete and abstract concepts. By contrast, the visually grounded vector of an abstract word is computed from the visual images of concrete words semantically associated with the abstract word, that is, *semantic neighbors* of the abstract word, assuming that semantic neighbors are good mediator words. As shown in Figure 1B where the word “love” is supposed to be abstract, its visually grounded vector $\vec{g}\left(\text{love}\right)$ is not computed directly from the images of “love,” but computed indirectly using the direct visual vectors, e.g., $\vec{v}\left(\text{kiss}\right)$, $\vec{v}\left(\text{mother}\right)$, $\vec{v}\left(\text{wedding}\right)$, derived from the images tagged with semantic neighbors (e.g., “kiss,” “mother,” and “wedding”). This computation is regarded as an implementation of indirect grounding for abstract concepts.

Formally, we define a multimodal distributional semantic model for indirect grounding as follows. We assume that the vocabulary *V* is divided into a set of concrete words *V*_*C*_ and a set of abstract words *V*_*A*_. Each word *w*_*i*_∈*V* has a textual vector $\vec{t}\left(w_{i}\right)\in DSM_{T}$ trained from a text corpus and a direct visual vector $\vec{v}\left(w_{i}\right)\in DSM_{V}$ computed directly from images for the word *w*_*i*_. We build an indirect grounding model *DSM*_*I*_ in which a word is represented by a pair $\left[\vec{t}\left(w_{i}\right),\vec{g}\left(w_{i}\right)\right]$ of a textual vector $\vec{t}\left(w_{i}\right)\in DSM_{T}$ and visually grounded vector $\vec{g}\left(w_{i}\right)\in DSM_{G}$. The visually grounded vector $\vec{g}\left(w_{i}\right)$ is defined as follows:


(1) $$\vec{g}(w_{i})\;=\;\{\begin{array}{ll} \vec{v}(w_{i}) & \left({\text{for a concrete word w}}_{\text{i}}\in {\text{V}}_{\text{C}}\right)\\ \frac{\sum_{w_{j}\in SN_{k}(w_{i})}\vec{v}(w_{j})}{k} & \left({\text{for an abstract word w}}_{\text{i}}\in {\text{V}}_{\text{A}}\right) \end{array}$$

where *SN*_*k*_(*w*_*i*_)⊂*V*_*C*_ is a set of *k* semantic neighbors of *w*_*i*_, that is, *k* concrete words most semantically related to *w*_*i*_. The visually grounded vector for an abstract word is thus obtained by averaging *k* direct visual vectors of semantic neighbors.

Semantic neighbors of abstract words are determined using word similarity in the text-based distributional semantic model *DSM*_*T*_. This implies that linguistic interdependency for indirect grounding is modeled by (text-based) distributional semantics. In the devised model, semantic neighbors *SN*_*k*_(*w*_*i*_)⊂*V*_*C*_ of an abstract word *w*_*i*_ are determined by first selecting *N*(>*k*) nearest neighbors of *w*_*i*_ from the whole vocabulary *V* and then selecting *k* nearest concrete words from the set of *N* neighbors. Nearest neighbors are computed using cosine similarity in the textual model *DSM*_*T*_. The reason for limiting *N* neighbors before selecting *k* concrete words is that some highly abstract words (e.g., “truth,” “wisdom”) may not have semantically related concrete words, and in this case it is more appropriate not to consider a visual representation. Hence, when no semantic neighbors are selected [i.e., *SN*_*k*_(*w*_*i*_) = ∅], no visual vector is considered and only the textual vector is used for representing the abstract word *w*_*i*_ in *DSM*_*I*_.

## 3. Materials and methods

### 3.1. Experimental design and predictions

**Figure 2:**
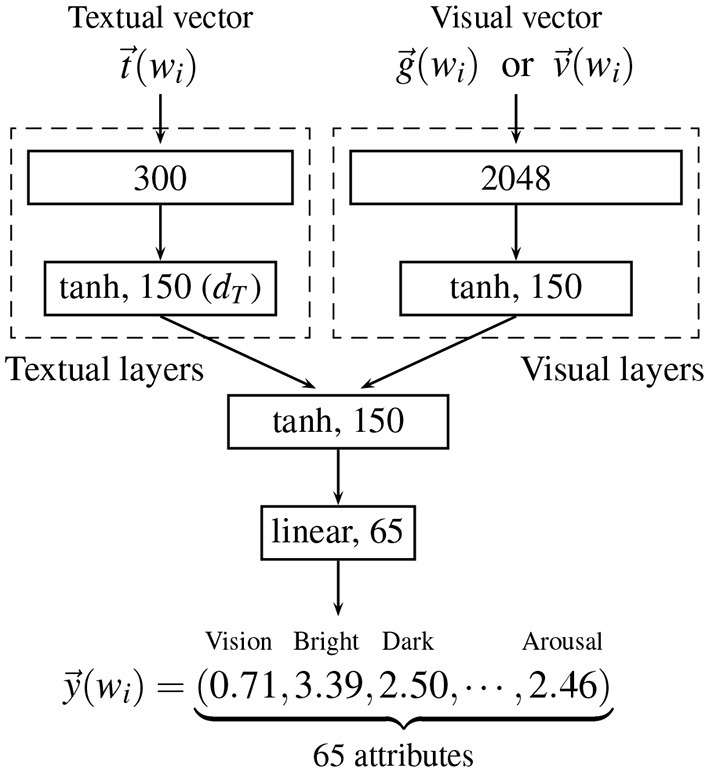
The neural network used for predicting Binder et al.'s (2016) conceptual representation $\vec{y}\left(w_{i}\right)$ of a word *w*_*i*_ from textual and/or visual vectors in the evaluation experiment. The visual vector used as input is either $\vec{g}\left(w_{i}\right)$ (for the indirect grounding and indirect visual models) or $\vec{v}\left(w_{i}\right)$ (for the hybrid, dual-coding, and visual models).

To evaluate the representational ability of a given distributional semantic model, we examined how accurately the model can predict human conceptual representation using Utsumi's (2020) experimental framework for analyzing and evaluating distributional semantic models. As human conceptual representation, we used a brain-based semantic representation provided by Binder et al. (2016), which is described in detail in section 3.2. In the evaluation experiment, a function from the bimodal (or unimodal) vector of a given word *w*_*i*_ (section 3.3) to the target human conceptual representation $\vec{y}\left(w_{i}\right)$ (section 3.2) was trained using the feed-forward neural network shown in Figure 2. The conceptual representation of untrained words was predicted by the trained neural network. Prediction performance was evaluated by comparing the estimated conceptual representation with the target representation. The details of training and test procedure is described in section 3.4.

To test whether the indirect grounding view is more plausible than other views on abstract concepts listed in Table 1, we conducted the evaluation experiment described above using the following distributional semantic models, that is, four models that correspond to each of the four views in Table 1 and two additional baselines.

- Indirect grounding model $DSM_{I}=\{[\vec{t}(w_{i}),\vec{g}(w_{i})]|\;\vec{t}(w_{i})\in DSM_{T},\vec{g}(w_{i})\in DSM_{G},w_{i}\in V\}$: A bimodal model for the indirect grounding view described in section 2.
- Hybrid model $DSM_{H}=\{[\vec{t}(w_{i}),\vec{v}(w_{i})]|\;\vec{t}(w_{i})\in DSM_{T},\vec{v}(w_{i})\in DSM_{V},w_{i}\in V\}$: A standard bimodal model in which all visual vectors are computed directly from images for a word *w*_*i*_. This model is assumed to simulate the hybrid view.
- Dual coding model $DSM_{D}=\{[\vec{t}(w_{i}),\vec{v}(w_{i})]|\;\vec{t}(w_{i})\in DSM_{T},\vec{v}(w_{i})\in DSM_{V},w_{i}\in V_{C}\}\;\cup \;\{\vec{t}(w_{i})|\;\vec{t}(w_{i})\in DSM_{T},w_{i}\in V_{A}\}$: A partially bimodal model in which a pair of textual and direct visual vectors is used for representing a concrete word, whereas only a textual vector is used for an abstract word. When abstract words are given in the training and test procedure, the visual layers in Figure 2 are not either trained or used. This model corresponds to the dual coding theory.
- Visual model $DSM_{V}=\left\{\vec{v}\left(w_{i}\right)|w_{i}\in V\right\}$: A unimodal model in which only direct visual vectors are used for representing words. Hence the textual layers in Figure 2 are not used in the training and test procedure. This model corresponds to the situated simulation view.
- Textual model $DSM_{T}=\left\{\vec{t}\left(w_{i}\right)|w_{i}\in V\right\}$: A unimodal baseline model in which only textual vectors are used for representing words. Hence the visual layers in Figure 2 are not used in the training and test procedure.
- Indirect visual model $DSM_{G}=\left\{\vec{g}\left(w_{i}\right)|w_{i}\in V\right\}$: A unimodal baseline model in which only indirect visual vectors (defined in Equation 1) are used for representing words. The textual layers in Figure 2 are ignored in the training and test procedure.

By comparing the prediction performance of the indirect grounding model with those of other models, we test the validity of indirect grounding as a plausible mechanism for embodied representation of abstract concepts. Different views of abstract concepts summarized in Table 1 make different predictions about the performance of the six models, as shown in Table 2. Basically, each of the four views predicts that its corresponding model would outperform other models. For abstract concepts, the dual coding theory predicts that the textual model also achieves the best performance because it argues that abstract concepts are represented only by linguistic information. For concrete concepts, the indirect grounding, hybrid, and dual coding views do not differ and thus make the same prediction that the indirect grounding, hybrid, and dual coding models do not significantly differ in performance and outperform the remaining models.

**Table 2:** Predictions of evaluation performance by conceptual representation theories.

	**Best (highest performance)**	
	**model predicted by each theory**	
**Theory**	**Abstract concepts**	**Concrete concepts**
Situated simulation view (e.g., Barsalou, 1999; Barsalou et al., 2018)	Visual	Visual
Dual coding theory citepPaivio71,Paivio86	Dual coding, textual	Dual coding, hybrid, indirect grounding
Hybrid view (e.g., Barsalou et al., 2008; Borghi et al., 2019)	Hybrid	Dual coding, hybrid, indirect grounding
Indirect grounding view (e.g., Howell et al., 2005; Louwerse, 2011)	Indirect grounding	Dual coding, hybrid, indirect grounding

### 3.2. Human conceptual representation

As a target human conceptual representation $\vec{y}\left(w_{i}\right)$, we used Binder et al.'s (2016) brain-inspired featural representation^2^. They provided 65-dimensional real-valued vectors of 535 words, some of which are listed in Table 3. These words comprise 434 nouns, 62 verbs, and 39 adjectives and are classified into 47 categories that reflect grammatical classes and semantic classes. Note that, for the reason explained later in section 3.3.3. two nouns were excluded from the experimental materials and thus the remaining 533 words were used in the evaluation experiment.

**Table 3:** Example of words included in Binder et al.'s (2016) dataset, which are selected mainly from abstract words.

**POS**	**Category**	**Word examples**
Noun	Abstract construct	analogy, irony, truth, verb, worth
	Cognitive entity	belief, hope, knowledge, sympathy, wit
	Emotion	gratitude, joy, love, shame, woe
	Social event	advice, deceit, matinee, snub, tribute
	Time period	day, era, evening, semester, summer
Verb	Locative action	approach, deliver, go, leave, walk
	Social action	arrest, celebrate, help, play, write
Adjective	Visual property	black, dark, new, red, shiny
	Emotional property	angry, dangerous, happy, lonely, peaceful

The dimensions of the vectors correspond to neurobiologically plausible attributes whose neural correlates have been well described. Table 4 lists all 65 attributes in 14 domains used in Binder et al.'s (2016) vectors. they correspond to distinguishable neural processors that can be identified by an extensive body of evidence from brain imaging and neurological studies, and they can contribute to concept acquisition and composition. Each value of the conceptual vectors represents the degree of salience of the corresponding attribute for the target word. Binder et al. (2016) collected these values using Amazon Mechanical Turk. The participants of the experiment were given a single word and questions such as “To what degree do you think of this thing as a characteristic or defining color (for the attribute *Color*)” with some examples, and asked to rate the degree on a 7-point scale ranging from 0 to 6. Collected ratings were averaged for each word and attribute after data screening, and these mean ratings were used in conceptual vectors.

**Table 4:** Sixty-five attributes used in Binder et al.'s (2016) conceptual representation.

**Domain**	**Attribute**
Vision	Vision, bright, dark, color, pattern, large, small, motion, biomotion, fast, slow, shape, complexity, face, body
Somatic	Touch, temperature, texture, weight, pain
Audition	Audition, loud, low, high, sound, music, speech
Gustation	Taste
Olfaction	Smell
Motor	Head, upper-limb, lower-limb, practice
Spatial	Landmark, path, scene, near, toward, away, number
Temporal	Time, duration, long, short
Causal	Caused, consequential
Social	Social, human, communication, self
cognition	Cognition
Emotion	Benefit, harm, pleasant, unpleasant, happy, sad, angry, disgusted, fearful, surprised
Drive	Drive, needs
Attention	Attention, arousal

### 3.3. Distributional semantic model

#### 3.3.1. Textual vector

Textual vectors $\vec{t}\left(w_{i}\right)\in DSM_{T}$ were trained on the Corpus of Contemporary American English (COCA), which included 0.56G word tokens. Words that occurred less than 30 times in the corpus were ignored, resulting in the training vocabulary of 108,230 words. As a distributional semantic model for training textual vectors, we used skip-gram with negative sampling (SGNS), which is one of two algorithms in word2vec model (Mikolov et al., 2013). In SGNS, a feed-forward neural network with one hidden layer of *d* units is trained to predict co-occurring words of an input word (i.e., *w* words appeared on either side of the input word in the corpus), and *d*-dimensional activation vectors in the hidden layer of the trained network are used as textual vectors. We set the vector dimension *d* = 300 and the window size *w* = 10. The choice of corpus, distributional semantic model, and parameter values was determined considering the result of the similar experiment (Utsumi, 2020).

#### 3.3.2. Visual vector

To compute direct visual vectors $\vec{v}\left(w_{i}\right)\in DSM_{V}$, we collected 20 images using *Flickr* image retrieval for each of the words in the vocabulary. The image retrieval was performed using the API flickr.photos.search with the argument sort=relevance and the top 20 most relevant images were downloaded for each word. Note that these relevant images are often tagged with other words, but we did not use the information of these tags.

To compute the feature vector of each downloaded image, we utilized the ResNet152-hybrid1365 model (Zhou et al., 2018)^3^. This model is the Residual Network (ResNet), which is a recent high-performance version of the deep convolutional neural networks, trained on both ImageNet1000 dataset for object recognition and Places365-standard dataset for scene recognition. Each image was entered into this model and a 2,048-dimensional activation vector was extracted from the last hidden layer. The activations in the last hidden layer are deemed to be appropriate for a visual vector in distributional semantics, because they are generally assumed to represent visually intrinsic features of a concept. Finally, the visual vector $\vec{v}\left(w_{i}\right)$ was computed as the centroid (i.e., average) of the activation vectors of 20 images.

#### 3.3.3. Indirect visual vector

To compute indirect visual vectors $\vec{g}\left(w_{i}\right)\in DSM_{G}$, we must determine how to split the whole vocabulary *V* into concrete words *V*_*C*_ and abstract words *V*_*A*_. For this purpose, we used Brysbaert et al.'s (2014) concreteness ratings for 39,354 English words including 37,058 single words and 2,896 two-word expressions. These words were rated on a 5-point scale ranging from 1 (abstract) to 5 (concrete) and the collected ratings were averaged per each word. In the instructions given to raters, Brysbaert et al. (2014) stressed that the assessment of word concreteness would be based on perceptual experiences involving all senses and motor responses. Specifically, the following instruction was used:

Some words refer to things or actions in reality, which you can experience directly through one of the five senses. We call these words concrete words. Other words refer to meanings that cannot be experienced directly but which we know because the meanings can be defined by other words. These are abstract words. (Brysbaert et al., 2014, p. 906)

This definition of concrete words as experience-based and abstract words as language-based is consistent with our view of abstract concepts, and thus the use of their concreteness ratings is appropriate for the indirect grounding model^4^.

For the vocabulary *V* in the indirect grounding model, we chose 28,437 words from Brysbaert et al.'s (2014) word concreteness dataset that were also included in the training vocabulary of COCA corpus and associated with at least 20 images. As a result, two words “ire” and “oration” in Binder et al.'s (2016) dataset were not included in the chosen vocabulary because they are not contained in Brysbaert et al.'s (2014) dataset. Hence, these two words were not used in the entire experiment.

Each word in the vocabulary *V* was judged as abstract if its concreteness rating was less than a given threshold θ_*c*_, and otherwise as concrete. We performed the same experiment with different thresholds ranging from 1.2^5^ to 5.0 with a step size of 0.1. In section 4, we report the overall result with a representative threshold θ_*c*_ = 3.0 at which any words whose concreteness rating is toward the language-based side of the continuum are classified as abstract. Additionally, we use the threshold θ_*c*_ = 4.0, at which words are treated as abstract unless they are rated as highly experience-based. Note that 116 and 214 out of 533 words in Binder et al.'s (2016) dataset (14,305 and 21,471 out of 28,437 words in the whole vocabulary *V*), respectively, were judged as abstract when θ_*c*_ = 3.0 and θ_*c*_ = 4.0.

After concrete words *V*_*C*_ and abstract words *V*_*A*_ are determined, indirect visual vectors $\vec{g}\left(w_{i}\right)$ are computed according to Equation (1). When a word *w*_*i*_ is concrete (i.e., *w*_*i*_∈*V*_*C*_), its direct visual vector $\vec{v}\left(w_{i}\right)$ defined in section 3.3.2 is used as a visual vector $\vec{g}\left(w_{i}\right)$. When a word *w*_*i*_ is abstract (i.e., *w*_*i*_∈*V*_*A*_), its semantic neighbors are determined using the textual vectors in section 3.3.1 as follows. First, *N*(>*k*) nearest neighbors of *w*_*i*_ are selected from the whole vocabulary *V* by computing cosine similarity between *t*(*w*_*i*_) and *t*(*w*_*j*_) for all words *w*_*j*_∈*V*(*j*≠*i*) and selecting words with top *N* highest cosine. Then, *k* nearest (i.e., highest cosine) concrete words are selected from the set of *N* neighbors. Finally, the direct visual vectors $\vec{v}\left(w_{j}\right)$ of *k* nearest neighbors are averaged and the resulting vector is used as $\vec{g}\left(w_{i}\right)$ for the abstract word *w*_*i*_.

### 3.4. Training and prediction

To train the mapping (i.e., prediction function) from bimodal (or unimodal) word vectors to Binder et al.'s (2016) conceptual representation, we used a feed-forward neural network shown in Figure 2. The activation function and the number of units are shown in each of the layers denoted by solid rectangles. The training was performed by minimizing the mean squared error (MSE), and gradient descent with Adam was used as an optimization method. The learning rate for Adam was fixed at 0.001. The weights (and biases) were initialized by the normalized initialization heuristic (Glorot and Bengio, 2010).

As an overall framework for evaluation (i.e., the procedure for training and prediction), we used a “leave-one-cluster-out” cross-validation procedure (Utsumi, 2020). This procedure is a variant of *n*-fold cross-validation in which semantic clusters for all words are used instead of randomly and equally partitioned groups. The reason for using leave-one-cluster-out cross-validation instead of *n*-fold cross-validation (and other methods with random sampling) is that words in Binder et al.'s (2016) dataset are not equally distributed in the semantic space. Some groups of words are semantically rich and they are very close to one another, whereas some other groups of words have only a small number of semantically less similar words. If we apply *n*-fold cross-validation to this dataset, semantically rich words with many close neighbors are likely to be better predicted independent of the representation ability of distributional semantic models, because their neighbors have more chance of being included in the training set.

To obtain word clusters for this procedure, we used Utsumi's (2020) method in which all 533 words were classified into 20 clusters using the k-means algorithm. Given textual vectors, we repeated k-means clustering 100 times and selected the best clustering result according to the Dunn index, which is a metric for evaluating clustering quality. Furthermore, to ensure the generality of the experimental results, we repeated this clustering procedure 10 times, and as a result, 10 different sets of 20 clusters were generated.

In the leave-one-cluster-out cross-validation procedure, for each cluster, the neural network in Figure 2 (i.e., prediction function) is trained using all words in the other clusters, and the conceptual vectors of words in the target cluster were predicted using the trained neural network. By repeating this procedure using each word cluster as a target, we obtained estimated conceptual vectors $\hat{\vec{y}}\left(w_{i}\right)$ for all 533 words. Prediction performance was measured by Pearson's correlation between the estimated vector $\hat{\vec{y}}\left(w_{i}\right)$ and the original vector $\vec{y}\left(w_{i}\right)$. Spearman's rank correlation ρ and MSE were also used as secondary measures. For each set of 20 clusters, this experimental run was carried out three times under the same condition (i.e., hyperparameters), and the result of the run with the highest mean correlation across all words was retained. Finally, the results obtained using 10 sets of clusters were averaged and used for the analysis reported in section 4.

Hyperparameters other than the concreteness threshold were determined using a grid search. First, we determined the number of epochs for training from hybrid, dual coding, visual, and textual models. Using the leave-one-cluster-out cross-validation, we computed MSE across all words with the number of epochs ranging from 1 to 50. The lowest MSE was obtained at 19 epochs for the hybrid model, 17 epochs for the dual coding and textual models, and 10 epochs for the visual model. For the indirect grounding model, two parameters *N* and *k* for computing semantic neighbors were optimized together with the number of epochs using grid search of *N* = 100, 200, 300 and *k* = 1, ⋯ , 10. In this grid search, we used the concreteness threshold θ_*c*_ = 4.0. Mean squared error was computed over all words and the lowest MSE was obtained at *N* = 300, *k* = 10, and 20 epochs. For the indirect visual model, we determined the number of epochs using indirect visual vectors at *N* = 300 and *k* = 10, and as a result, 16 epochs achieved the lowest MSE. These hyperparameters were used in all experimental runs for evaluation.

## 4. Results

### 4.1. Performance difference among models

**Figure 3:**
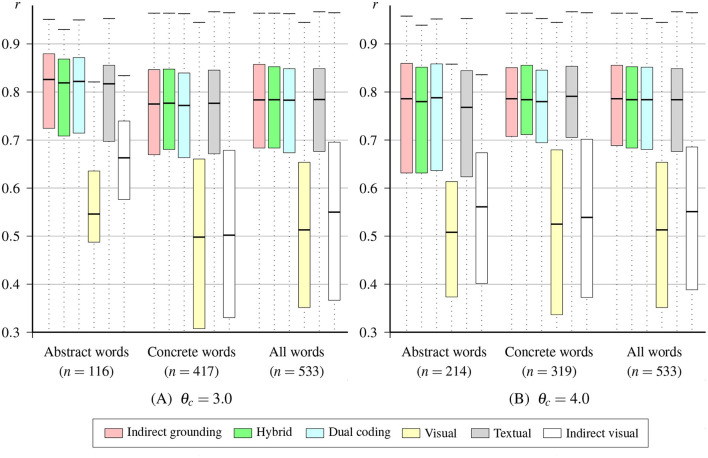
Boxplots of word correlations for the indirect grounding model and other models.

Table 5 lists mean correlations between the original conceptual vector and the vectors estimated by the indirect grounding model and other models. Figure 3 shows the variation in correlation coefficients over abstract, concrete, and all words. Note that in this section we report the results of Pearson's correlation when used as performance measure, but we also analyzed the performance with two additional measures (i.e., Spearman's rank correlation and MSE). Because these results do not significantly differ from those of Pearson's correlation, the detailed results of the additional analysis are provided in Appendix S1 of the Supplementary material.

**Table 5:** Mean correlations for the indirect grounding model and other models.

	θ_*****c*****_ = 3.0			θ_*****c*****_ = 4.0		
**Model**	**Abstract**	**Concrete**	**All**	**Abstract**	**Concrete**	**All**
**Bimodal**			
Indirect grounding (*DSM*_*I*_)	**0.772**	0.742	**0.749**	**0.731**	0.761	**0.749**
Hybrid (*DSM*_*H*_)	0.764	**0.744**	0.748	0.724	**0.764**	0.748
Dual coding (*DSM*_*D*_)	0.762	0.734	0.740	0.729	0.756	0.745
**Unimodal**			
Visual (*DSM*_*V*_)	0.536	0.475	0.488	0.480	0.494	0.488
Textual (*DSM*_*T*_)	0.755	0.740	0.744	0.716	0.762	0.744
Indirect visual (*DSM*_*G*_)	0.626	0.490	0.520	0.529	0.513	0.519

Boldfaced numbers indicate the highest correlations (i.e., the best performance) among the models.

For abstract words, the indirect grounding model *DSM*_*I*_ achieved the highest mean correlation in both concreteness thresholds. The Friedman test conducted on abstract words revealed a significant difference between word correlations of six models, χ^2^(5, *N* = 116) = 280.20, *p* < 0.001 for θ_*c*_ = 3.0 and χ^2^(5, *N* = 214) = 583.23, *p* < 0.001 for θ_*c*_ = 4.0. Multiple pairwise comparisons using the Wilcoxon signed-rank test with Ryan's procedure (*p* < 0.05) showed that the correlation of the indirect grounding model was significantly higher than those of all other models at θ_*c*_ = 3.0 and than those of other four models except the dual coding model at θ_*c*_ = 4.0. For other pairwise differences, only the difference between the dual coding and hybrid models was not significant for either thresholds. This result is consistent with the prediction of the indirect grounding view in Table 2. Additionally, the result that the indirect visual model *DSM*_*G*_ predicted the conceptual representation better than the simple visual model *DSM*_*V*_ also indicates the effectiveness of computing visual vectors using semantic neighbors. These results clearly support the indirect grounding view of abstract concepts.

Although it is not the main concern of this paper whose focus lies in abstract concepts, the Friedman test conducted on correlations of concrete words also indicated a significant difference among six models, χ^2^(5, *N* = 417) = 1181.66, *p* < 0.001 for θ_*c*_ = 3.0 and χ^2^(5, *N* = 319) = 895.97, *p* < 0.001 for θ_*c*_ = 4.0. The highest mean correlation was achieved by the hybrid model for both concreteness thresholds, but multiple pairwise comparison revealed that pairwise differences among the hybrid, indirect grounding, and textual models were not significant. For θ_*c*_ = 4.0, the difference between the dual coding and textual models and between the dual coding and indirect grounding models also did not reach the significance level. All the other pairwise differences were significant. The absence of significant difference among the indirect grounding, hybrid, and dual coding models is a predictable result, as shown in Table 2. What is somewhat surprising is that bimodal models for abstract concepts (i.e., the indirect grounding and hybrid models) did not achieve significantly higher performance than the textual (i.e., unimodal) model, given that a number of studies on multimodal distributional semantics have shown the superiority over text-based unimodal models for concrete words (e.g., Bruni et al., 2014; Baroni, 2016). This result is not consistent with the prediction of Table 2 made by the indirect grounding, hybrid, and dual coding views.

One possible reason would be that the textual layer may cover most of the information needed to predict the target conceptual representation of concrete words; the textual layer compresses 300-dimensional input textual vectors to 50% (= 150/300) of their original dimension, but the visual layer compresses 2,048-dimensional input visual vectors to a much lower percent, 7.3% (= 150/2, 048). To test this possibility, we conducted an additional experiment with the same experimental procedure by decreasing the dimension *d*_*T*_ of the textual (hidden) layer. The detailed result of this additional experiment is provided in Appendix S2 of the Supplementary material. The result is supportive of this possibility; when the dimension *d*_*T*_ was 30 (whose compression rate 10.0% is nearly equal to that of the visual layer) or lower, the bimodal models (i.e., indirect grounding and hybrid models) achieved significantly higher correlations than the unimodal textual model. Note also that decreasing the dimension *d*_*T*_ of the textual layer did not affect the result of abstract words; the same result of performance differences were obtained regardless of *d*_*T*_. From these results, it follows that the visual layer actually contributes to model performance in an expected way and our bimodal distributional semantic model achieves a result fully consistent with the prediction of the indirect grounding view when the impact of the textual and visual layers is equalized.

In addition, the difference among six models was also significant for all words, χ^2^(5, *N* = 533) = 1438.50, *p* < 0.001 for θ_*c*_ = 3.0 and χ^2^(5, *N* = 533) = 1444.01, *p* < 0.001 for θ_*c*_ = 4.0. The difference between the indirect grounding and hybrid models at θ_*c*_ = 3.0 and the differences among the indirect grounding, hybrid, and dual coding models at θ_*c*_ = 4.0 were not significant, but all the other pairwise comparisons were significant.

Summarizing, the obtained results are most consistent with the predictions of the indirect grounding view shown in Table 2. It is therefore concluded that the indirect grounding view is plausible as a conceptual representation theory of abstract concepts.

### 4.2. Effect of the concreteness threshold

**Figure 4:**
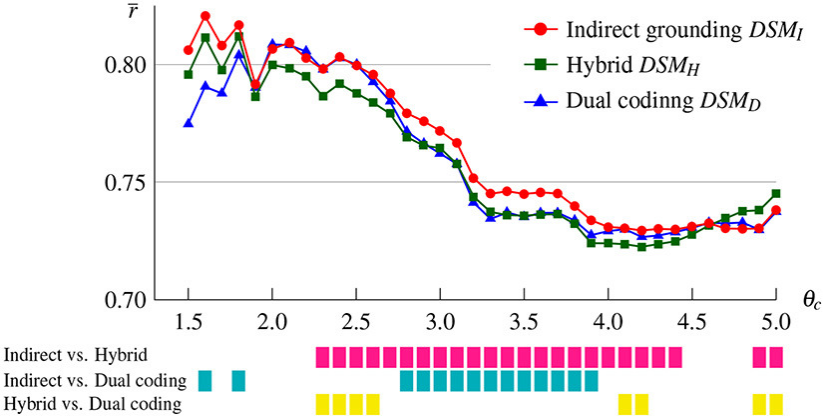
Mean correlations over abstract words for the indirect grounding, hybrid, and dual coding models as a function of the concreteness threshold θ_*c*_. Pairwise differences significant at *p* < 0.05 are indicated by color bars below the graph.

To test whether the superiority of the indirect grounding model for abstract concepts reported in the last section holds for other concreteness thresholds, we conducted the same experiment at different thresholds θ_*c*_ ranging from 1.5 to 5.0 with a step size of 0.1. Figure 4 shows mean correlations over abstract words for the indirect grounding, hybrid, and dual coding models. Color bars shown below the graph denote whether pairwise differences were statistically significant by multiple pairwise comparison (*p* < 0.05).

The indirect grounding model yielded a higher correlation than other two models when the concreteness threshold was between 1.5 and 4.5, although at some lower threshold the correlation of the indirect grounding model was slightly lower than that of the dual coding model. Specifically, the correlation of the indirect grounding model was significantly higher than that of the hybrid model between θ_*c*_ = 2.3 and 4.4, and than that of the dual coding model between θ_*c*_ = 2.8 and 3.9. This confirms the finding reported in section 4.1 and indicates that the superiority of the indirect grounding model was not accidentally observed in some concreteness thresholds. At θ_*c*_ = 4.6 or higher, the mean correlation of the indirect grounding model was lower than the hybrid model. In these cases, highly concrete words were selected as abstract and their visual vectors were computed via semantic neighbors, even though it is appropriate that they are grounded directly through their own visual images. This “less plausible” grounding may generate a harmful effect on the prediction performance of the indirect grounding model. This behavior of the model is also consistent with the indirect grounding view.

### 4.3. Relation between word concreteness and improvement by indirect grounding

In this section, we examine whether the performance improvement of the indirect grounding model compared to the hybrid and dual coding models depends on word concreteness. To quantify the degree of improvement, we considered the difference of correlation computed by subtracting the correlation coefficient of a baseline model from the correlation coefficient of the indirect grounding model.

We computed a correlation between the difference of correlation and word concreteness. The difference of correlation was not correlated with word concreteness when the hybrid model is a baseline, *r* = −0.104 (θ_*c*_ = 3.0) and *r* = 0.075 (θ_*c*_ = 4.0). In the case of the dual coding model used as a baseline, the difference of correlation was not correlated with word concreteness for θ_*c*_ = 4.0, *r* = 0.071, but they were weakly correlated for the threshold θ_*c*_ = 3.0, *r* = 0.222 (*p* < 0.05). These results indicate that there was generally no monotonic relationship between the degree of improvement and word concreteness, but appending indirect visual vectors to textual vectors may be somewhat more effective for less abstract concepts.

**Figure 5:**
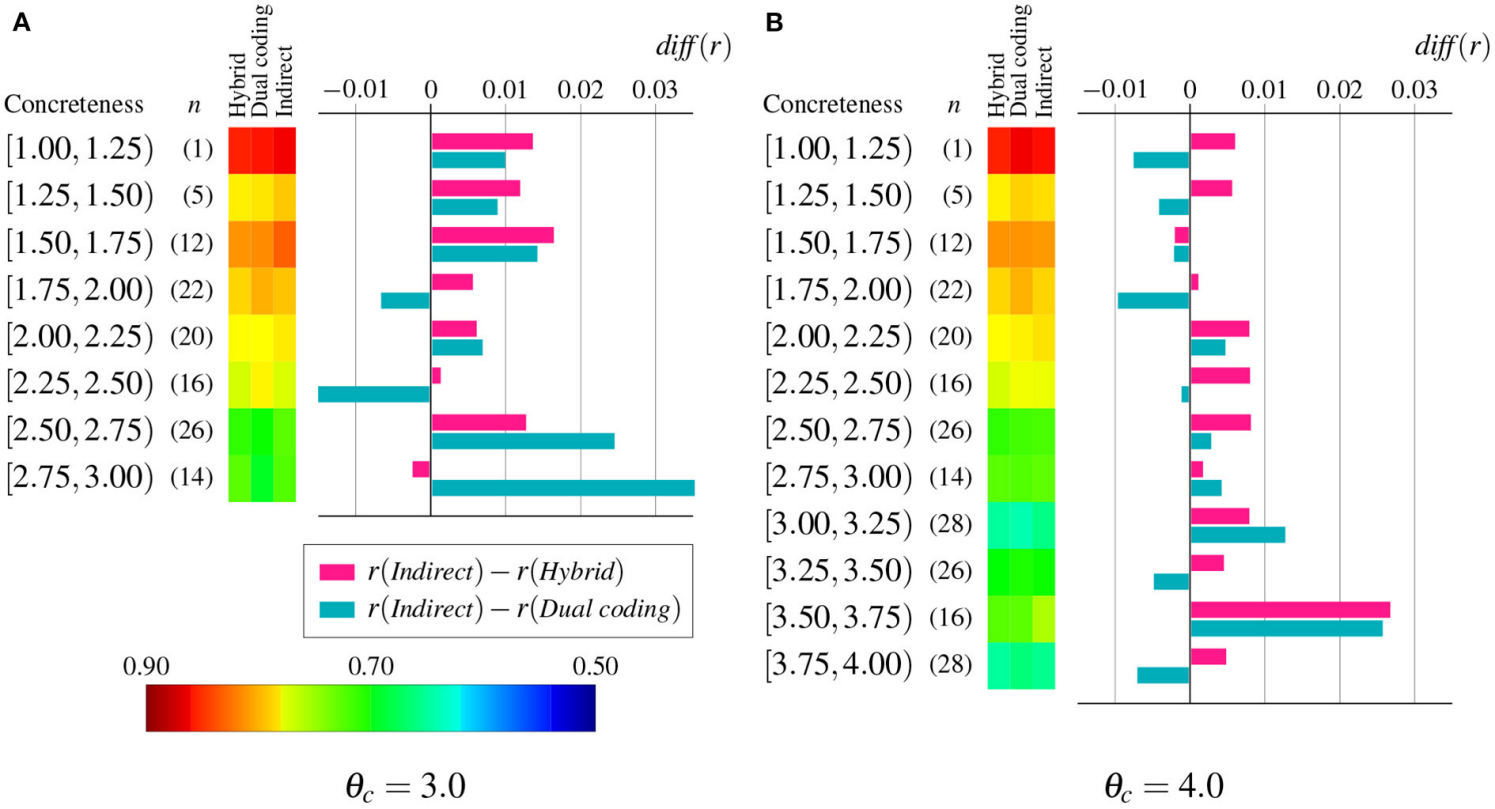
Mean difference of correlation *diff*(*r*) between the indirect grounding model and two competing models per each of the equally divided intervals of concreteness. The heatmap depicts the mean correlation of hybrid, dual coding, and indirect grounding models. Numbers in parentheses denote the number of words *n* within an interval. Red and blue graphs, respectively, denote the degree of improvement against the hybrid model and the dual coding model. **(A)** θ_*c*_ = 3.0. **(B)** θ_*c*_ = 4.0.

To examine more closely the relation between performance improvement and word concreteness, we also computed the mean difference of correlation per each of the intervals into which the entire range of concreteness values was equally divided, whose results are shown in Figure 5. Overall, the indirect grounding model improved the prediction performance regardless of word concreteness, but two concreteness thresholds showed different patterns of improvement. For θ_*c*_ = 3.0, the indirect grounding model improved the performance for highly abstract words (i.e., words with concreteness rating is less than 1.75) against both competing models. This result suggests that the indirect grounding model is effective in representing purely abstract words. Furthermore, only when comparing with the dual coding model, the degree of improvement was higher for less abstract words (i.e., the ones within the range of 2.50 ≤ concreteness rating < 3.00) than for more abstract words. This may suggest that these words, which may be difficult to judge concreteness or have both concrete and abstract senses, benefit from adding visual information whether directly or indirectly.

For θ_*c*_ = 4.0, however, the performance of highly abstract words (whose concreteness rating was less than 2.0) were not improved and the difference of correlation peaked at higher concreteness range [3.50, 3.75]. These different results for highly abstract words between both concreteness thresholds can be attributed primarily to the difference of semantic neighbors, because θ_*c*_ determines a word pool (i.e., a set of concrete words) from which semantic neighbors are chosen. Hence the effectiveness of indirect grounding depends on not only the concreteness of a word to be grounded, but also the choice of semantic neighbors for that word.

### 4.4. Word-level analysis on the impact of indirect grounding

Abstract (and concrete) concepts have been considered as a unitary whole, but recent research has argued that abstract concepts should be treated as a heterogeneous category including various different types of abstract concepts (Ghio et al., 2016; Troche et al., 2017; Borghi et al., 2018; Villani et al., 2019). Therefore, to examine the types of abstract concepts for which indirect grounding, in particular, visually indirect grounding, is effective, we analyzed the degree of improvement in terms of semantic categories of abstract words.

**Figure 6:**
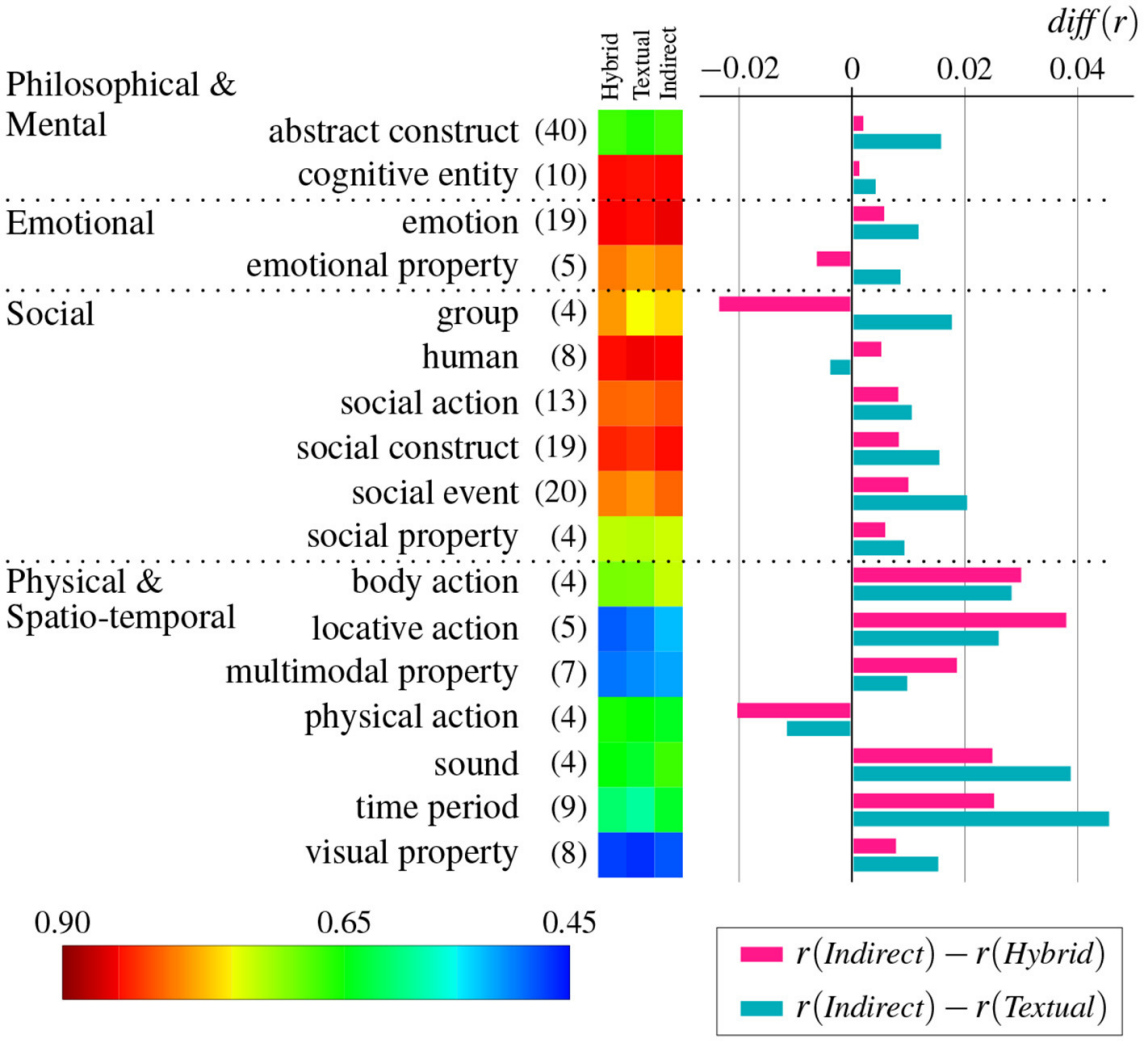
Mean difference of correlation *diff*(*r*) between the indirect grounding model and two baseline models averaged per semantic category for abstract words (θ_*c*_ = 4.0). The heatmap depicts the mean correlation of hybrid, textual, and indirect grounding models. Numbers in parentheses denote the number of abstract words contained in semantic categories. Red and blue graphs, respectively, denote the degree of improvement against the hybrid model and the textual model.

Figure 6 shows how the degree of improvement is related to semantic categories for abstract words with concreteness rating less than 4.0 (i.e., θ_*c*_ = 4.0). We used semantic categories provided by Binder et al. (2016) to classify abstract words. They classified all 535 words into 47 categories that reflect semantic and grammatical classes. Among them, we selected 17 categories for analysis that included four or more abstract words. In addition, these 17 categories were grouped according to the following four clusters revealed by Villani et al. (2019): physical, spatio-temporal, and quantitative (*Physical and Spatio-temporal*) concepts, self and sociality (*Social*) concepts, philosophical/spiritual (*Philosophical and Mental*) concepts, and emotional/inner states (*Emotional*) concepts.

**Figure 7:**
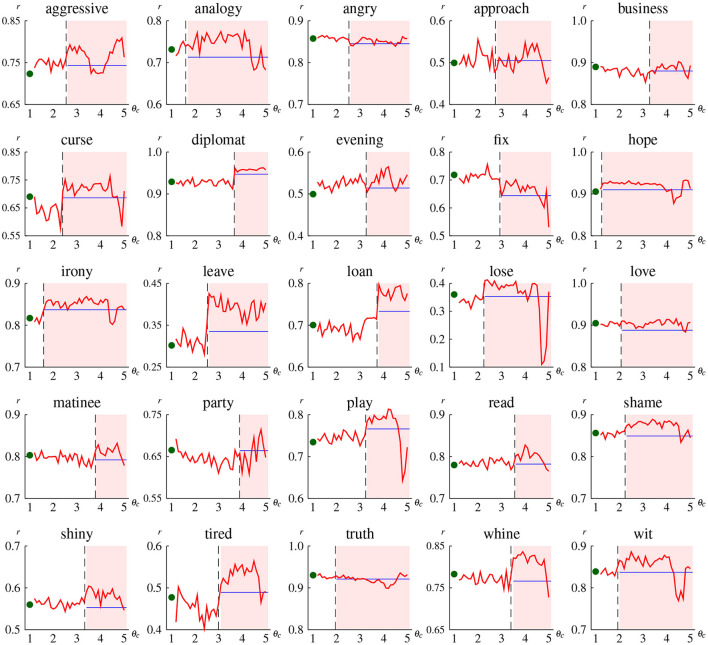
Correlation coefficient of the indirect grounding model *DSM*_*I*_ for some individual abstract concepts as a function of concreteness threshold θ_*c*_. The plot at θ_*c*_ = 1.0 denotes the correlation of the hybrid (i.e., direct grounding) model *DSM*_*H*_ (because *DSM*_*I*_ with θ_*c*_ = 1.0 is identical to *DSM*_*H*_). The blue horizontal line is drawn at the correlation coefficient of the textual model *DSM*_*T*_. The dashed vertical line denotes the word concreteness, and thus the line chart in the red shaded area represents the correlation obtained using indirect visual vectors.

As shown in Figure 6, *Social* and *Physical and Spatio-temporal* clusters are likely to show higher improvement than *Philosophical and Mental* and *Emotional* clusters. In particular, *body action, locative action, sound*, and *time period* categories in *Physical and Spatio-temporal* cluster achieved relatively higher improvement by the indirect grounding model. The high improvement of physical and spatio-temporal concepts is consistent with the indirect grounding view, because these abstract concepts are likely to be associated (via language) with specific visual images. For example, the concept *evening* easily evokes visual experiences associated with the concepts of *dinner* and *sunset*. In the experiment of this paper, “night,” “dinner,” “supper,” “dusk,” “dawn,” “sundown,” “twilight,” “candlelight,” “sunset,” and “sunrise” were selected as semantic neighbors of the word “evening,” and the indirect visual vectors computed from these images improved the baselines as shown in Figure 7. The relatively high improvement of social categories can be explained along the same line. A number of social concepts are associated with perceptually grounded concepts. For example, the verb *play* can be captured by relevant concepts such as *game, soccer* and *football* (objects to be played) and *sandlot* (place to play), which were selected as semantic neighbors. Meanwhile, some other social concepts (e.g., *business* and *joke*) are more complex and difficult to capture by grounded concrete concepts.

By contrast, emotional categories were not improved by the indirect grounding model. Emotional information is highly likely to be encoded in textual vectors (e.g., Recchia and Louwerse, 2015; Utsumi, 2020), and thus indirect grounding may not be necessary for emotional concepts. A more plausible explanation would be that emotional concepts are directly grounded in emotional experiences, and thus relatively less dependent on indirect grounding in perceptual (i.e., visual) experiences. The lesser degree of improvement for the cluster of *Philosophical and Mental* is not surprising. These abstract concepts are generally thought of as “highly disembodied” concepts (Dove, 2016), which are divorced from experiential (at least visual) grounding regardless of whether the grounding is direct or indirect.

Figure 7 shows the change of correlation coefficient in terms of concreteness threshold for some abstract words. The result of all abstract words with concreteness rating less than 4.0 is provided in Appendix S3 of the Supplementary material. The important point to note is the difference of correlation between non-shaded and shaded areas. Correlation plotted in the red shaded area (i.e., the region right of the dashed vertical line) shows the prediction performance of the indirect grounding model when a word is regarded as abstract and thus indirect visual vectors are used, whereas correlation in the non-shaded area shows the performance when that word is regarded as concrete and direct visual vectors are used. Hence, the indirect grounding model is found to be effective for words whose correlations in the shaded area are higher than those in the non-shaded area. A typical pattern in this effective case is that correlations in the shaded area are higher than those in the non-shaded area as well as higher than those of textual and hybrid models (e.g., “evening,” “leave,” “play”). This pattern is also marked by the decrease of correlation at higher thresholds (i.e., θ_*c*_≥4.5), because good mediator words for an abstract word are erroneously judged as abstract and thus no longer selected as semantic neighbors at higher thresholds. By contrast, some words (e.g., “angry,” “business,” “truth”) show a different pattern that correlation does not largely differ between the shaded and non-shaded area, which indicates no performance improvement by the indirect grounding model. For some other words (e.g., “fix”), correlation decreases in the shaded area; this pattern indicates that the indirect grounding model is harmful to predicting conceptual representation.

## 5. Discussion

### 5.1. Contribution to the research on abstract concepts

The present study makes an original contribution to research on abstract concepts. As mentioned in section 1, very few empirical studies have demonstrated direct evidence in favor of the indirect grounding view, although a number of studies have empirically shown that both symbolic/linguistic and perceptual/embodied representations are required for shaping and processing abstract concepts. Given the current lack of direct evidence, the present study provides empirical support specific to the role of language posited by the indirect grounding view. The higher prediction performance of the indirect grounding model compared to the hybrid (i.e., direct grounding) model suggests that a mere combination of symbolic and perceptual representation (e.g., Barsalou et al., 2008) is less adequate for explaining abstract concepts (or at least those that can be grounded in visual experiences); abstract concepts are more likely to be indirectly grounded through their linguistic relations to the concepts directly grounded in the world. In other words, language functions as a bridge between abstract concepts and perceptual experiences.

The plausibility of indirect grounding is also supported by the result of comparing two visual models. Even when textual vectors were not used for prediction, the indirect visual model outperformed the simple visual model, as reported in section 4.1. This implies that the use of visual vectors derived from concrete words strongly associated with an abstract word stands on its own merit.

Furthermore, the superiority of the indirect grounding model over two unimodal models (i.e., textual and visual models) and the dual coding model suggests that abstract concepts are both linguistic and grounded in perceptual experience. This result is consistent with the recent empirical findings (Louwerse and Jeuniaux, 2010; Malhi and Buchanan, 2018) and thus lends further support to the hybrid views of abstract concepts (Louwerse, 2011; Dove, 2016; Borghi et al., 2019).

### 5.2. Related work on computational approaches to indirect grounding

Some existing studies have proposed a computational model for semantic processing that is based on similar views to indirect grounding. (mentioned in section 1.3) using a simple recurrent network. The network was trained to predict, from the current input word, both what the next word would be and the featural (i.e., sensorimotor) representation of the current word. They demonstrated that the network trained with featural representation achieved better performance in next word prediction than the network trained without featural representation, and argued that this result supports the propagation of grounding. However, their model does not directly simulate the process in which the sensorimotor features of concrete concepts are propagated to abstract concepts. assimilates a very similar idea to indirect grounding; “Knowledge of abstract words is acquired through (a) their patterns of co-occurrence with other words and (b) acquired embodiment, whereby they become indirectly associated with the perceptual features of co-occurring concrete words (Hoffman et al., 2018, p. 293).” Their model is based on a hub-and-spoke architecture in which the information of an input word, its sensorimotor properties and past states (as context) is integrated into a hidden “hub” layer. They trained the model to predict the next word in a word sequence and showed that the trained model could represent the semantic knowledge of concrete and abstract words in a hub layer and accounted for behavioral patterns consistent with normal and impaired semantic cognition. However, they did not quantitatively test whether indirect grounding is more plausible than other competing views, such as one that abstract concepts are also grounded directly in sensorimotor experience.

These previous studies differ from the present study in some important respects. First of all, they use, as perceptual or sensorimotor representation, only verbally expressed featural information, which is essentially symbolic and discrete. Second, they do not directly test the plausibility of indirect grounding for representing abstract concepts; their proposed models are not compared with other competing models to be considered. Furthermore, their studies are limited in their coverage of the vocabulary of words and features; only a relatively small set of words and features are used in the experiments. The training corpus is also small in size and generated artificially. By contrast, the present study directly uses non-verbal (i.e., visual) information as perceptual representation and quantitatively tests the indirect grounding view by comparing other competing models including the direct grounding model. The vocabulary and corpus used in the experiment of the present study are relatively large.

To the best of our knowledge, no prior studies on multimodal distributional semantics or other computational models using non-verbal information have tested indirect grounding of abstract concepts (or words), but a noteworthy observation was reported. by extending the objective function of the original skip-gram (Mikolov et al., 2013) so as to take into account visual similarity computed using visual vectors. Using the trained multimodal word vectors, they showed that some abstract words had nearest neighbors in the trained multimodal space whose visual images depict relevant concrete situations (e.g., the nearest neighbor picture of the word “theory” depicts a bookshelf with many books), although the nearest neighbors of many other abstract words were not visually relevant. The extended skip-gram model does not directly simulate the process of indirect grounding, but this result suggests that the multimodal skip-gram may be a useful model for exploring the grounding mechanism of abstract concepts.

### 5.3. Limitation of this study and future direction

Our distributional semantics-based approach to embodied cognition of abstract concepts has its limitations. One important limitation is that the method for modeling perceptual experiences used in this paper does not deal with perceptual information other than visual one. Although people are supposed to acquire a large percentage of information from visual perception, conceptual knowledge is also grounded in other types of perceptual experiences, such as auditory, somatosensory, gustatory, and olfactory ones, as well as from emotional and social experiences (e.g., Borghi et al., 2017). Our finding in favor of indirect grounding is thus confined to visual grounding; the detailed analysis based on semantic categories reported in section 4.4 revealed that abstract concepts in only some categories (i.e., *Social* and *Physical and Spatio-temporal* categories) benefit from visually indirect grounding. Future research is required to investigate whether and how other types of abstract concepts are grounded directly or indirectly.

A more noteworthy limitation is that the DNN by which visual vectors are computed may diverge from human visual perception. The progress of deep learning technique has demonstrated that DNNs surpass human-level performance on some specific image classification tasks (Zhang et al., 2021) and it has been shown that their internal representations match coarsely with the brain (Cichy et al., 2016; Serre, 2019). By contrast, recent studies have also revealed that DNNs show behavioral deviations from human visual perception, for example, in terms of the sensitivity to global shape (Baker et al., 2018) and the visual representational structure in the human brain (Xu and Vaziri-Pashkam, 2021). Jacob et al. (2021) demonstrated that some phenomena (e.g., surface invariance, sensitivity to 3D shape) seen in human visual perception were not observed in ResNet-152, which is used for extracting visual vectors in this study, as well as in other DNNs. These deviations suggest that DNNs and its visual vectors are limited as a cognitive model of visual grounding. Given these potential limitations, we must be cautious about interpreting the obtained results, in particular the lower performance of the visual model *DSM*_*V*_ as evidence against the situated simulation view.

Our choice of text-based distributional semantic model (i.e., SGNS) by which textual vectors are computed is unlikely to greatly affect the obtained results, given the current technical possibilities. that is, SGNS, GloVe (Pennington et al., 2014), and PPMI+SVD (Bullinaria and Levy, 2007), in terms of performance in predicting Binder et al.'s (2016) conceptual representation only from textual vectors. He demonstrated that SGNS achieved the highest prediction performance, and more importantly, the relative performance differences among words and attributes were quite similar among three models. of textual models using the same prediction task. These models include BERT (Devlin et al., 2018), which is a deep neural model for contextual embeddings that achieves state-of-the-art performance in many NLP tasks. They reported that BERT did not show any significant differences from the distributional semantic models analyzed by Utsumi (2020), and word and attribute correlation of BERT is equivalent to that of SGNS. These results imply that the obtained findings in this paper have certain generality with respect to textual models. Obviously, however, it does not mean that current language models sufficiently capture semantic (or conceptual) knowledge people can acquire from language (for a review, see Rogers et al., 2020; Lake and Murphy, 2021). If a psychologically more plausible language model is developed in the future, it would be interesting to explore whether the indirect grounding model still yields the same result.

The present study also suffers from methodological limitations. Binder et al.'s (2016) dataset used as a target conceptual representation for the evaluation experiment is the most comprehensive and fine-grained featural representation publicly available at present, but yet not sufficient to capture the richness of human conceptual knowledge. For example, concepts (particularly abstract concepts) generally involve the knowledge of binary and multiary relations among concepts and higher-order relations that cannot be expressed by feature-based representation. Hence the obtained result does not reflect the representational ability of relational knowledge. Furthermore, the training procedure for predicting Binder et al.'s (2016) representation poses an additional concern about whether it can precisely capture processing differences among competing models. This concern is particularly salient for the dual coding model. According to its definition in section 3.1, the dual coding model should approximate the performance of the textual model for abstract words and that of the hybrid model for concrete words. However, the result of section 3.1 (Appendix S2) diverged from these expectations; the dual coding model outperformed the textual model in predicting abstract words and showed lower performance for concrete words than the hybrid model. This discrepancy between expectations and performance may be caused by simultaneous training of concrete and abstract words. Specifically, network parameters (i.e., weights and biases) between the output layer (i.e., the bottom layer of Figure 2) and the hidden layer just above reflect the visual information of concrete words, even when visual vectors are not given. Because of this, prediction of an abstract word may indirectly reflect visual information of concrete concepts whose textual vectors are similar to that of the abstract word, and thus the dual coding model would perform better than the textual model. For concrete words, the dual coding model does not benefit from visual information for abstract words and this may cause lower performance of the dual coding model than the hybrid model. Although this discrepancy does not affect the findings on the superiority of the indirect grounding model, more valid training procedures should be pursued.

The use of concreteness rating may be controversial because of its limitations. Most concreteness ratings including Brysbaert et al.'s (2014) are collected by presenting words in isolation, thereby including an ambiguity in judgment for polysemous words (e.g., Reijnierse et al., 2019). This is particularly problematic for words with both concrete and abstract senses, which tend to be rated around the middle of the scale. In other words, words with low concreteness ratings are likely to be unambiguous and regarded as definitely abstract. The analysis reported in section 4.3 showed that the indirect grounding model improved the performance for words with low concreteness raging (when appropriate semantic neighbors were given), thus suggesting that our finding in favor of the indirect grounding view does not severely affected by this problem of concreteness rating.

Despite the positive result, there is another problem with the use of concreteness rating that must be addressed in future work. Recent studies on abstract concepts have argued that abstract concepts are not a unitary whole and should be treated as a heterogeneous category including various different types of abstract concepts (Troche et al., 2017; Borghi et al., 2018; Villani et al., 2019). This argument implies that concreteness rating is not sufficient for determining words (or concepts) to be grounded indirectly. As reported in section 4.4, the impact of indirect grounding differs among various types of abstract concepts. Exploring this issue may provide an interesting avenue for future investigation.

The choice of semantic neighbors as mediator concepts, whose visual vectors define the indirectly grounded representation of abstract words, is an important process of the indirect grounding model. Appropriate mediator concepts need to have perceivable referents that also have perceptually clear-cut boundaries. For example, basic-level concepts such as *desk* and *chair* have specific referents that are characterized by perceptual features such as shapes, and thus they become good mediators. Superordinate concepts such as *furniture* also have perceivable referents but are difficult to distinguish from other concepts by perceptual features, and thus they are less likely to be good mediators. Highly underspecified concepts such as *artifact* no longer function as mediators because they are very generic and their defining features are not based on perceptual or other bodily experiences. However, our simple method of generating a pool of candidate words from which semantic neighbors are chosen has a potential problem in that words with higher concreteness rating do not necessarily have such specific referents. For example, the word “furniture” has a higher concreteness rating of 4.89 than “desk” (4.87) and “chair” (4.58), and the word “artifact” also has a very high rating of 4.50 in Brysbaert et al.'s (2014) dataset. Recently Bolognesi et al. (2020) empirically examined the same line of argument and demonstrated that categorical specificity should be considered as a distinct dimension from concreteness to characterize concepts. Categorical specificity is therefore an important property for choosing appropriate mediator words, although an appropriate level of specificity depends on the concept to be indirectly grounded.

Some other methods for determining mediator words can be considered to refine the indirect grounding model. Age-of-acquisition ratings (Kuperman et al., 2012) may be used to limit the vocabulary of candidate words for semantic neighbors, because basic words learned at the early stage of lexical acquisition are represented primarily perceptually, while other words learned at the later stage are acquired through the knowledge of basic words (Gleitman et al., 2005; Thill et al., 2014). A more promising approach is to use minimal grounding sets (Vincent-Lamarre et al., 2016) as a candidate set of semantic neighbors. A minimal grounding set is the smallest set of words (i.e., a subset of a vocabulary) from which all the other words in a vocabulary can be defined. Vincent-Lamarre et al. (2016) proposed a method for computing the minimal grounding sets from dictionary definitions. Although the minimal grounding set is not uniquely determined, it can be a theoretically more motivated pool of potential mediator words than a set of concrete words simply selected based on concreteness rating or other human-rating-based measures. Image tags may also be a useful source of information for mediator words because tag co-occurrence statistics reflect visually motivated semantic knowledge. For example, words that are similar to a target abstract word in Flickr distributional tagspace (Bolognesi, 2017) are expected to be good mediators.

## 6. Conclusion

To test the indirect grounding view, we devised a new multimodal distributional semantic model in which a visual vector of an abstract word (i.e., embodied representation of perceptual experiences) is computed from the visual images of concrete words semantically related to the abstract word. Through the evaluation experiment, we have demonstrated that the indirect grounding model outperformed the hybrid (i.e., direct grounding) model, the dual coding model, and unimodal models. Despite the limitations described above, this finding lends some plausibility to the indirect grounding view and the present study is regarded as a first step toward empirically exploring the grounding mechanism of abstract concepts. For future work, we would like to explore a further mechanism of what and how linguistic processes come into play for grounding abstract concepts, together with to test indirect grounding via language using psychological experiments.

## Data availability statement

The raw data supporting the conclusions of this article will be made available by the authors, without undue reservation.

## Author contributions

AU conceptualized the study, performed the evaluation experiment and statistical analysis, and wrote the draft of the manuscript.

## Funding

This research was supported by JSPS KAKENHI Grant Numbers JP15H02713 and JP20H04488.

## Conflict of interest

The author declares that the research was conducted in the absence of any commercial or financial relationships that could be construed as a potential conflict of interest.

## Publisher's note

All claims expressed in this article are solely those of the authors and do not necessarily represent those of their affiliated organizations, or those of the publisher, the editors and the reviewers. Any product that may be evaluated in this article, or claim that may be made by its manufacturer, is not guaranteed or endorsed by the publisher.

## Footnotes

^1^As explained in section 3, the task used in the experiment of this study is to predict a featural representation of concepts comprising attributes and their salience. This task can be regarded as a kind of deeper conceptual processing such as a property generation task that requires both linguistic and simulation (i.e., embodied) systems.

^2^Binder et al.'s (2016) data is available at http://www.neuro.mcw.edu/semanticrepresentations.html.

^3^The ResNet152-hybrid1365 model is publicly available at https://github.com/CSAILVision/places365.

^4^Furthermore, although Brysbaert et al. (2014) instructed that word concreteness would be judged according to experiences involving all five senses, the analysis on the obtained concreteness ratings revealed that participants largely focused on visual and haptic experiences. This result further justifies the appropriateness of their data for using to determine abstract words for our model focusing only on visual experience.

^5^We did not use θ_*c*_ = 1.1 because the minimum concreteness rating in Binder et al.'s (2016) dataset was 1.19 (for the word “belief”) and no words were judged as abstract when θ_*c*_ = 1.1.
