# Supplementary material for: A test of indirect grounding of abstract concepts using multimodal distributional semantics
Source: Front Psychol. 2022 Oct 4;13:906181. doi: 10.3389/fpsyg.2022.906181 (PMC9577286; doi:10.3389/fpsyg.2022.906181)
Supplement: Supplementary file 1 [file Data_Sheet_1.pdf]

A Test of Indirect Grounding of Abstract Concepts Using Multimodal Distributional Semantics

Supplementary Material

Akira Utsumi
Department of Informatics & Artificial Intelligence eXploration Research Center
The University of Electro-Communications
1-5-1 Chofugaoka, Chofushi, Tokyo 182-8585, Japan
utsumi@uec.ac.jp

A Test of Indirect Grounding of Abstract Concepts
Using Multimodal Distributional Semantics

Supplementary Material

Appendix S1: Model performance measured by Spearman's rank correlation and mean squared error

Appendix S1 reports the additional results of the evaluation experiment obtained by using Spearman's rank correlation and mean squared error (MSE) as performance measures.

Rank correlation

Table S1 lists mean rank correlations between the original target conceptual vector and the vectors estimated by the indirect grounding model and other models.

For abstract words, the indirect grounding model achieved the highest rank correlation at the concreteness threshold $\theta_c = 3.0$. The Friedman test conducted on abstract words showed a significant difference among word correlations of six models, $\chi^2(5, N = 116) = 184.27$, $p < .001$. Multiple pairwise comparisons using the Wilcoxon signed-rank test with Ryan's procedure ($p < .05$) showed that the indirect grounding model had significantly higher correlations than all other models. Only pairwise differences among the hybrid, dual coding, and textual models were not significant. In the case of $\theta_c = 4.0$, the rank correlation of the indirect grounding model was slightly lower than that of the dual coding model, but higher than those of other four models. The Friedman test showed a significant difference of correlations among six models, $\chi^2(5, N = 214) = 451.72$, $p < .001$, and all pairwise differences except between the indirect grounding and dual coding models were statistically significant ($p < .05$). These results are equivalent to those reported in the main article (i.e., those obtained using Pearson's correlation used as a performance measure) and most consistent with the prediction of the indirect grounding view.

For concrete words, the Friedman test also indicated a significant difference of correlations among six models, $\chi^2(5, N = 417) = 1015.67$, $p < .001$ for $\theta_c = 3.0$ and $\chi^2(5, N = 319) = 749.92$, $p < .001$ for $\theta_c = 4.0$. The highest mean correlation was achieved by the textual model for both concreteness thresholds, but multiple pairwise comparisons showed that pairwise differences among the textual, indirect grounding, and hybrid models were not significant. The difference between the indirect grounding and dual coding models also did not reach the significance level. All the other pairwise differences were significant. These results for concrete words are also equivalent to those reported in the main article.

Table S1

Mean rank correlations for the indirect grounding model and other models.

Model	$\theta_c = 3.0$			$\theta_c = 4.0$		
	Abstract	Concrete	All	Abstract	Concrete	All
Bimodal						
Indirect grounding (DSM_I)	**.724**	.709	**.7124**	.698	.724	**.713**
Hybrid (DSM_H)	.716	.711	.7118	.690	.726	.711
Dual coding (DSM_D)	.712	.704	.706	**.699**	.719	.711
Unimodal						
Visual (DSM_V)	.556	.482	.498	.502	.495	.498
Textual (DSM_T)	.708	**.712**	.711	.683	**.729**	.711
Indirect visual (DSM_G)	.634	.490	.521	.554	.511	.528

Note. Boldfaced numbers indicate the highest correlations (i.e., the best performance) among the models.

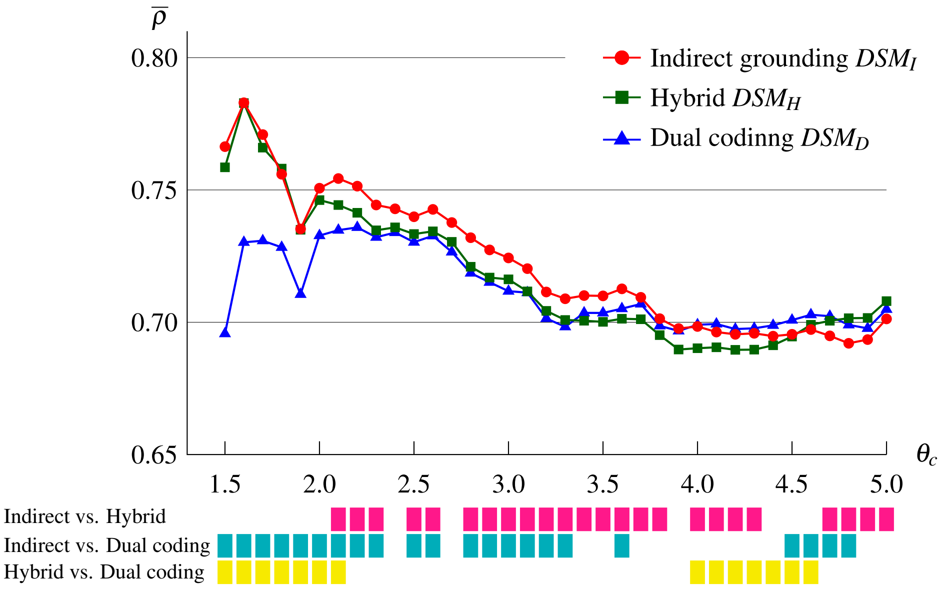

Figure S1. Mean rank correlations over abstract words for the indirect grounding, hybrid, and dual coding models as a function of the concreteness threshold θ_c. Pairwise differences significant at $p < .05$ are indicated by color bars below the graph.

When the rank correlation of all words was entered into the Friedman test, there was a significant difference among six models, $\chi^2(5, N = 533) = 1164.19$, $p < .001$ for $\theta_c = 3.0$ and $\chi^2(5, N = 533) = 1174.36$, $p < .001$ for $\theta_c = 4.0$. The pairwise differences among the indirect grounding, hybrid, and textual models were not significant for both concreteness thresholds, and the dual coding model also did not differ from these three models at $\theta_c = 4.0$.

Figure S1 depicts mean rank correlations over abstract words for the indirect grounding, hybrid and dual coding models at different concreteness thresholds θ_c ranging from 1.5 to 5.0 with a step size of 0.1. The overall tendency does not differ from the result of Pearson's correlation shown in Figure 4 of the main article. The indirect grounding model yielded a higher rank correlation than the hybrid model when the concreteness threshold θ_c is between 1.5 and 4.5, and at most of these threshold values the difference of correlation was significant. The indirect grounding model also yielded a higher rank correlation than the dual coding model at $\theta_c = 3.9$ or lower. In particular, a significantly higher correlation than the dual coding model was observed more frequently at lower thresholds than in the case of Pearson's correlation.

Mean squared error

Table S2 lists mean MSEs between the original target conceptual vector and the vectors estimated by the six models.

For abstract words, the Friedman test conducted on MSE showed a significant difference between word correlations of six models, $\chi^2(5, N = 116) = 246.41$, $p < .001$ for $\theta_c = 3.0$ and $\chi^2(5, N = 214) = 534.14$, $p < .001$ for $\theta_c = 4.0$. The indirect grounding model achieved the lowest MSE at $\theta_c = 3.0$ and multiple pairwise comparisons showed that the indirect grounding model had significantly lower MSE than all other models ($p < .05$). Only the difference between the hybrid and dual coding models was not significant. In the case of $\theta_c = 4.0$, the indirect grounding model did not yield lower MSE than the dual coding model, but the difference between the two models was not significant. All other pairwise differences were significant. These results of MSE are equivalent to those of Pearson's correlation reported in the main article and of rank correlation reported above. Hence, it can be concluded that the result of the evaluation experiment supports the indirect grounding view regardless of performance measure.

For concrete words, the Friedman test also indicated a significant difference of MSE among six models, $\chi^2(5, N = 417) = 1114.87$, $p < .001$ for $\theta_c = 3.0$ and $\chi^2(5, N = 319) = 835.60$, $p < .001$ for $\theta_c = 4.0$. The lowest MSE was achieved by the hybrid

Table S2

Mean MSEs for the indirect grounding model and other models.

	$\theta_c = 3.0$			$\theta_c = 4.0$		
Model	Abstract	Concrete	All	Abstract	Concrete	All
Bimodal						
Indirect grounding (DSM_I)	**0.741**	1.077	1.004	0.871	1.098	1.006
Hybrid (DSM_H)	0.765	**1.069**	**1.003**	0.891	**1.078**	1.003
Dual coding (DSM_D)	0.776	1.110	1.037	**0.855**	1.100	**1.002**
Unimodal						
Visual (DSM_V)	1.381	1.877	1.769	1.556	1.912	1.769
Textual (DSM_T)	0.803	1.089	1.026	0.925	1.095	1.026
Indirect visual (DSM_G)	1.156	1.865	1.711	1.447	1.892	1.713

Note. Boldfaced numbers indicate the lowest MSE (i.e., the best performance) among the models.

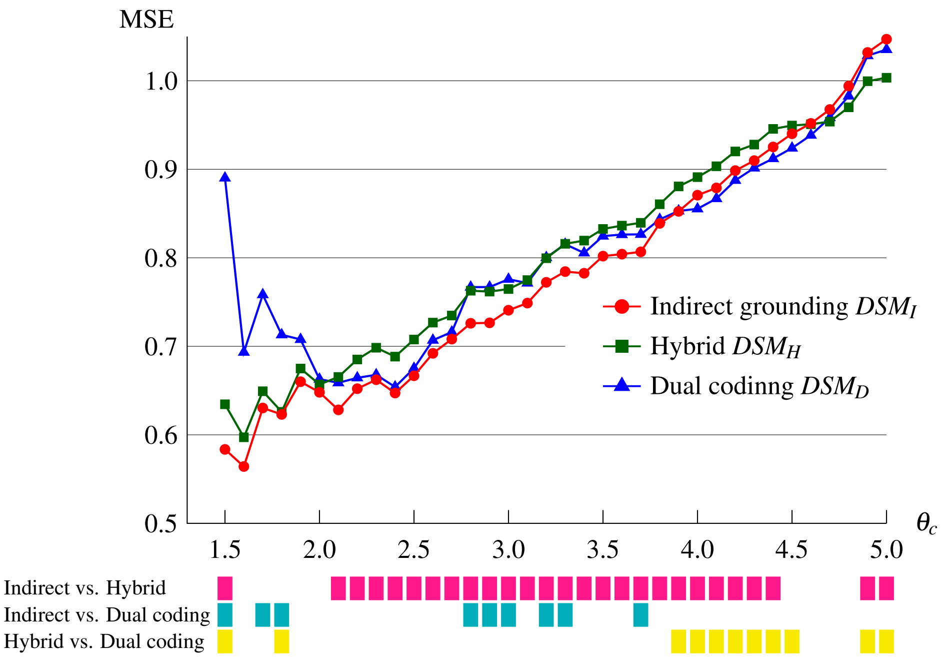

Figure S2. Mean MSEs over abstract words for the indirect grounding, hybrid, and dual coding models as a function of the concreteness threshold θ_c. Pairwise differences significant at $p < .05$ are indicated by color bars below the graph.

model for both concreteness thresholds, but multiple comparisons showed that pairwise differences among the hybrid, indirect grounding, and textual models were not significant. The difference between the dual coding model and these three models also did not reach the significance level for $\theta_c = 4.0$. In addition, the difference between the visual model and the indirect visual model was not significant for both thresholds. All the other pairwise differences were significant. These results are almost equivalent to those of two correlation measures.

When the MSE of all words was entered into the Friedman test, there was a significant difference among six models, $\chi^2(5, N = 533) = 1322.59$, $p < .001$ ($\theta_c = 3.0$) and $\chi^2(5, N = 533) = 1337.69$, $p < .001$ ($\theta_c = 4.0$). For $\theta_c = 3.0$, the hybrid model yielded the lowest MSE but did not significantly differ from the indirect grounding model. For $\theta_c = 4.0$, the dual coding model yielded the lowest MSE, but the pairwise differences between the dual coding, indirect grounding, and hybrid models were not significant.

Figure S2 shows mean MSEs over abstract words for the indirect grounding, hybrid and dual coding models at different concreteness thresholds θ_c ranging from 1.5 to 5.0 with a step size of 0.1. The overall tendency does not differ from the result of Pearson's and Spearman's correlation. The indirect grounding model achieved lower MSE than the hybrid model when the concreteness threshold θ_c is between 1.5 and 4.5, and the difference of MSE was significant at most of these threshold values. The indirect grounding model also achieved lower MSE than the dual coding model when θ_c is between 1.5 and 3.9, but the range of threshold value where the difference was significant was narrower than in the case of Pearson's and Spearman's correlation.

Appendix S2: Does the visual layer actually contribute to model performance?

The result of the main experiment showed that prediction performance of concrete words did not significantly differ between the bimodal models and the unimodal textual model, which is not consistent with either theoretical prediction (Table 1) or empirical findings on multimodal semantic models (Section 1.4). This unexpected result may be caused because the textual layer encodes language information enough to predict Binder et al.'s (2016) conceptual representation of concrete words, and thus the effect of visual layer, if any, may be hidden. To test this hypothesis, we conducted the same experiment as the main article by repeatedly decreasing the dimension d_T of the textual layer from 140 to 10 in steps of 10 and lastly at $d_T = 5$. If the bimodal models achieve higher performance than the unimodal textual model by the lower dimensions of the textual layer, it follows that our bimodal distributional semantic model is valid and thus our experimental results are reliable.

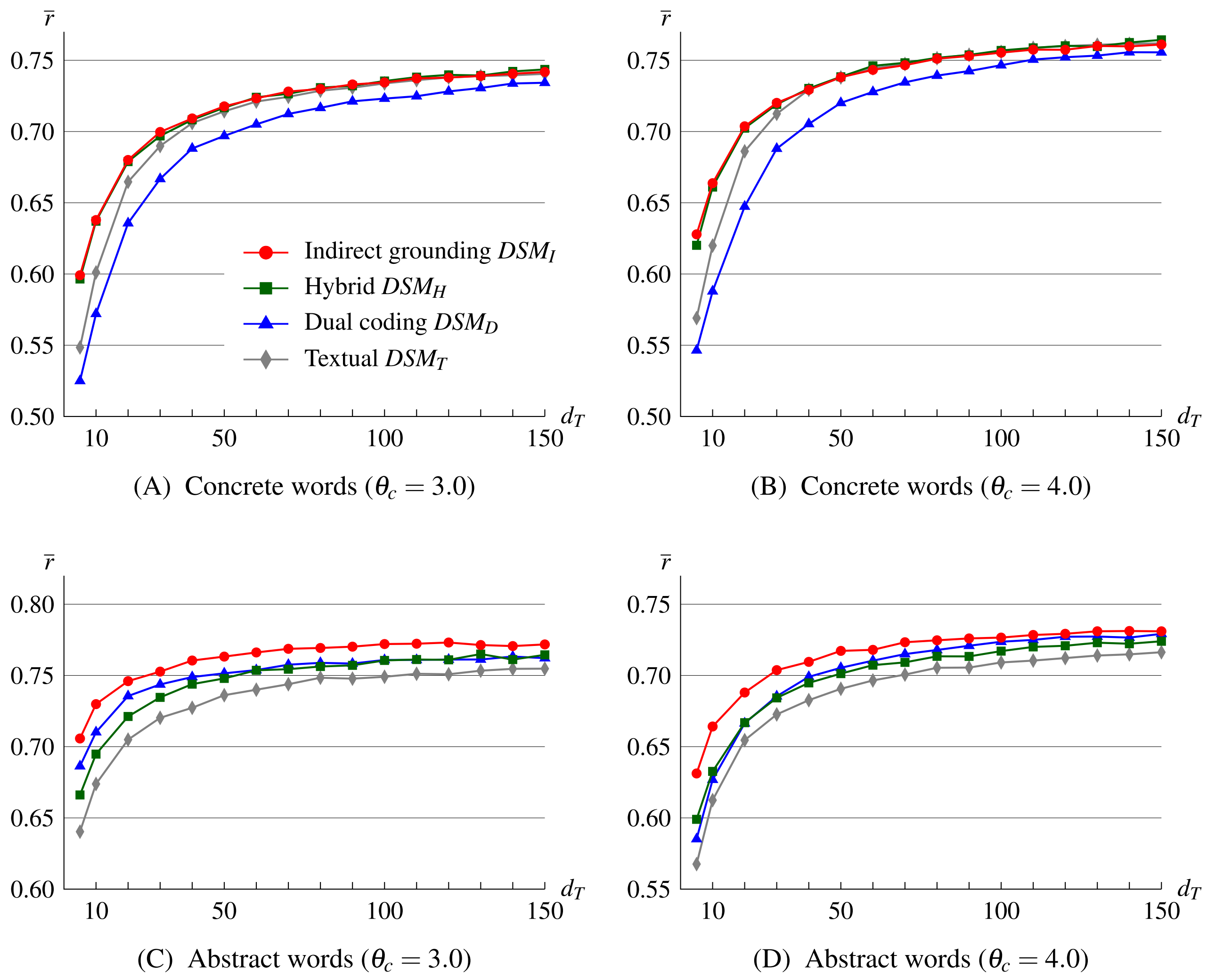

Figure S3. Mean correlations over concrete and abstract words as a function of the hidden dimension d_T of the textual layer.

Figures S3 (A) and (B) show mean correlations over concrete words when the concreteness threshold θ_c is 3.0 and 4.0. For both concreteness thresholds, the indirect grounding and hybrid models achieved clearly higher correlation than the textual model at the dimension d_T was 30 or lower. Furthermore, multiple pairwise comparisons using the Wilcoxon signed-rank test ($p < .05$) showed that these differences of correlation between the bimodal models and the textual model were statistically significant. For $\theta_c = 3.0$, the correlation of the indirect grounding model was also significantly higher than that of the textual model at $d_T = 40$. These results indicate that, when the compression rate of the textual layer (i.e., 0.100 for $d_T = 30$) was nearly equal to or lower than that of the visual layer (i.e., 0.073), the visual information of our bimodal distributional semantic models has a theoretically expected impact on the prediction of target conceptual representation.

We also note that, as shown in Figures S3 (C) and (D), the result of abstract words was

replicated even when the textual dimension d_T was decreased. The indirect grounding model achieved the highest correlation for abstract words, regardless of the dimension of the textual layer and concreteness threshold. As a result, even when the impact of the textual and visual information is equalized, our indirect grounding model yielded the results fully consistent with the prediction of the indirect grounding view.

Appendix S3: Prediction results of all individual abstract words

The following graphs, some of which are shown in Figure 7 of the main body of this paper, show the correlation coefficients of the indirect grounding model DSM_I as a function of concreteness threshold θ_c for all 214 abstract words whose concreteness rating is less than 4.0. The plot at $\theta_c = 1.0$ denotes the correlation of the hybrid (i.e., direct grounding) model DSM_H (because DSM_I with $\theta_c = 1.0$ is identical to DSM_H). The blue horizontal line is drawn at the correlation coefficient of the textual model DSM_T. The dashed vertical line denotes the word concreteness, and thus the line chart in the red shaded area represents the correlation obtained using indirect visual vectors. The graphs are grouped according to the categories provided by Binder et al. (2016).

- Abstract action

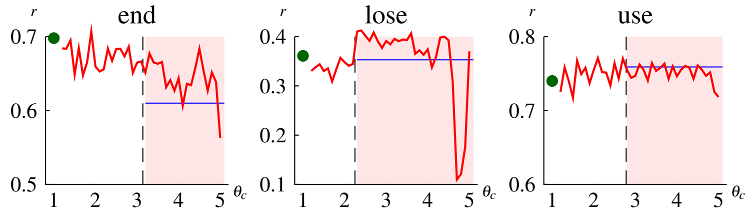

- Abstract construct

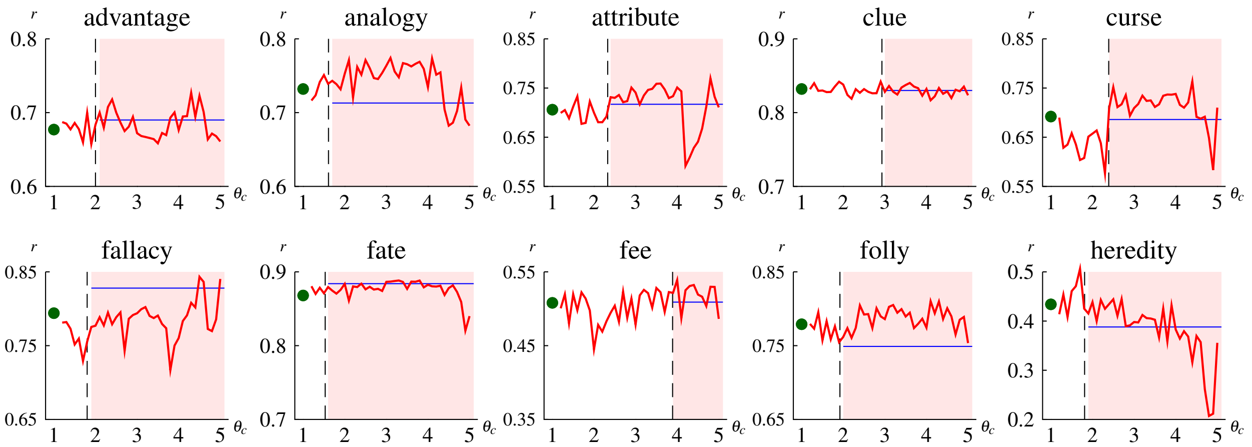

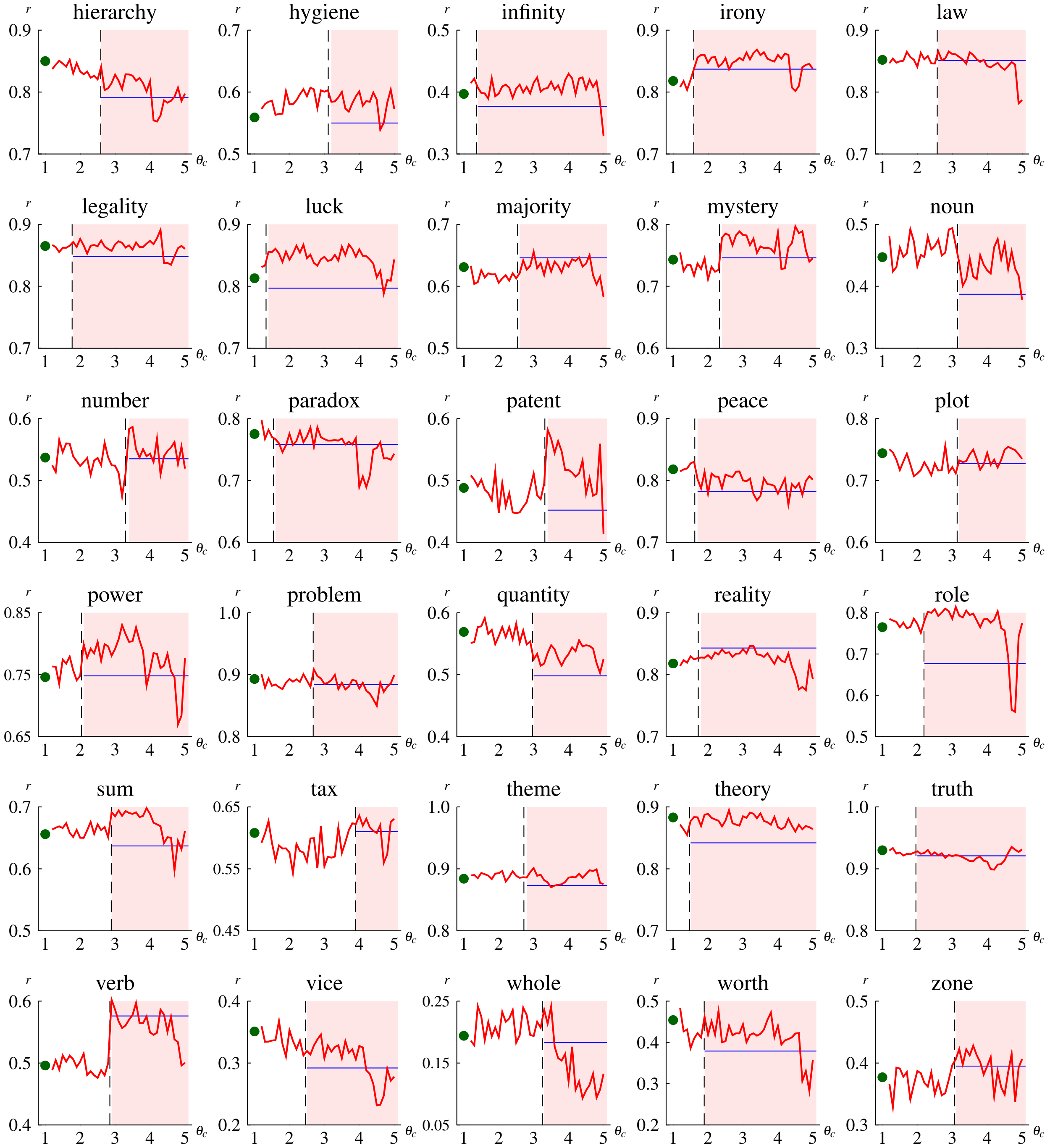

- Auditory property

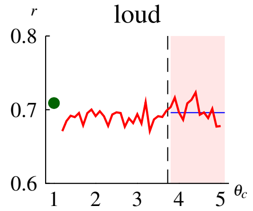

- Body action

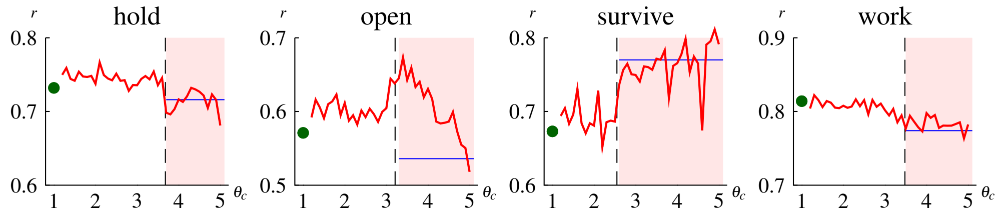

- Body state

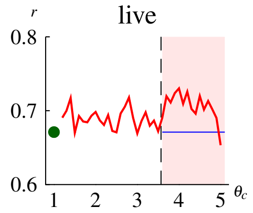

- Cognitive entity

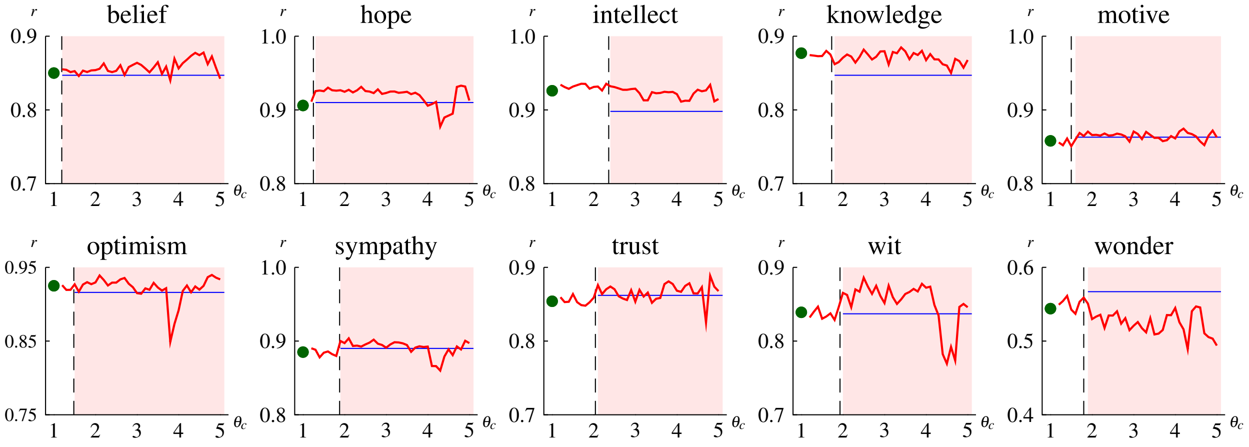

- Cognitive property

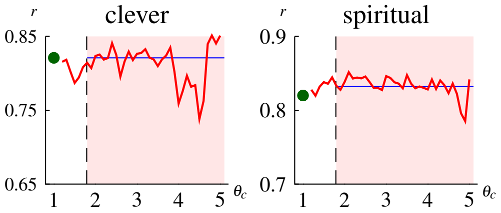

- Creative action

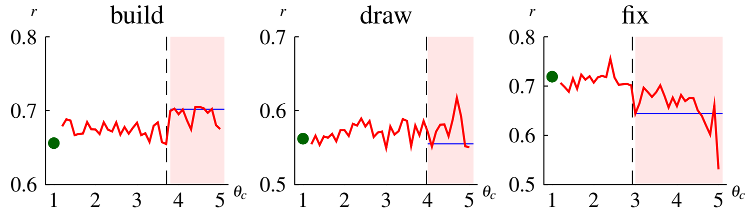

- Document

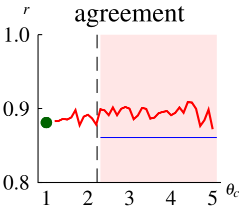

- Emotion

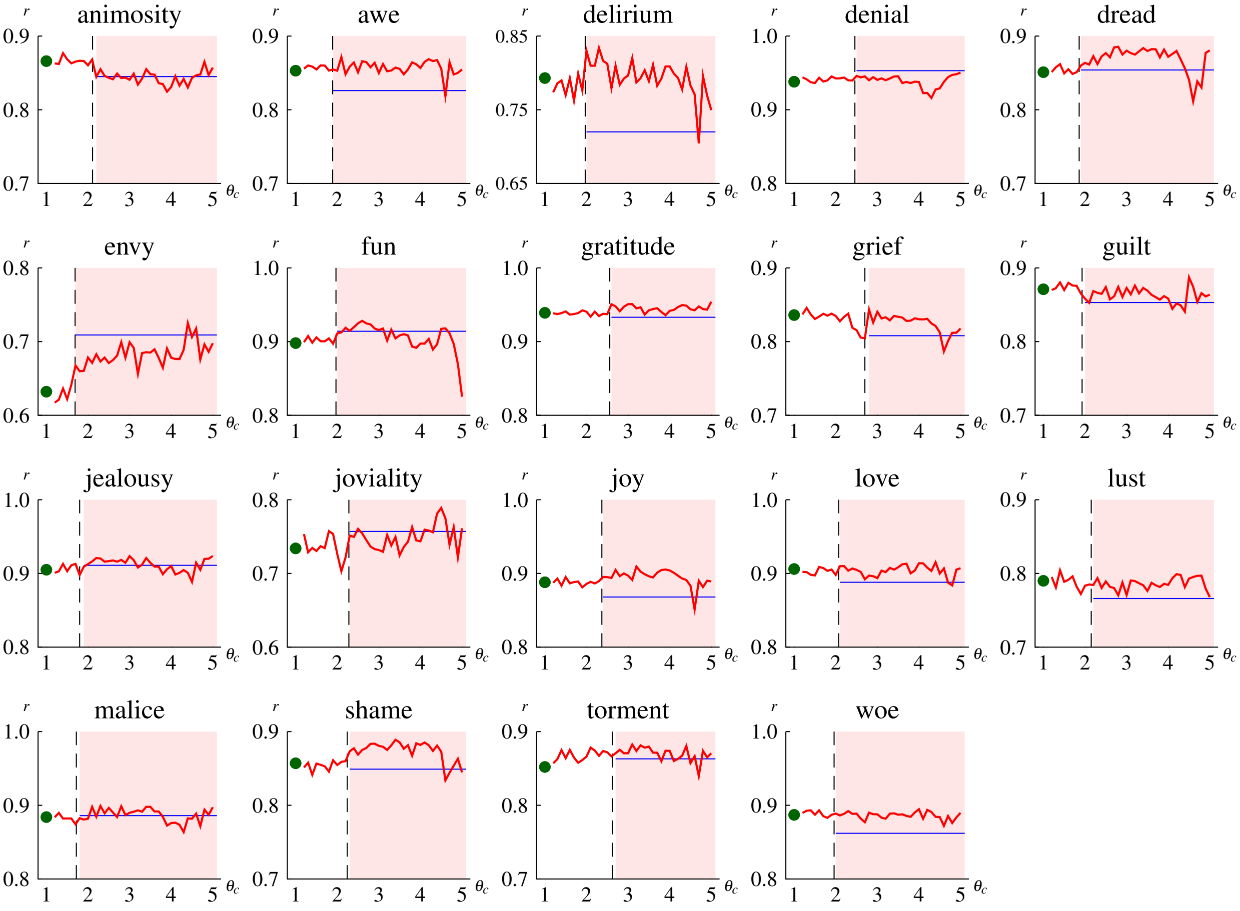

- Emotional property

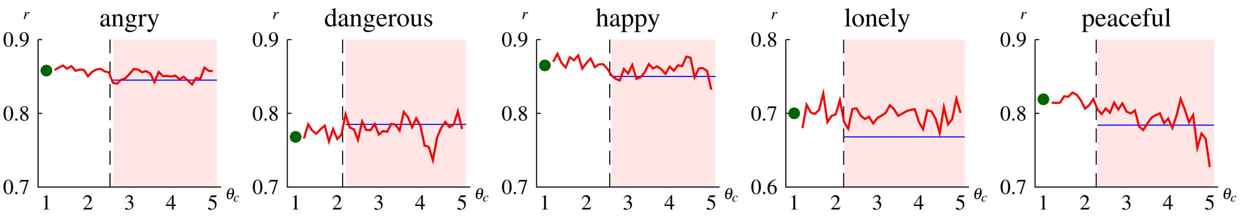

- General event

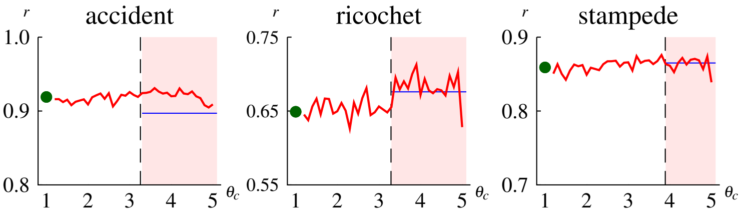

- Group

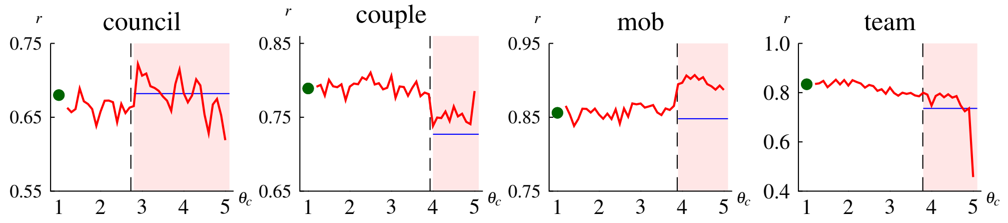

- Human

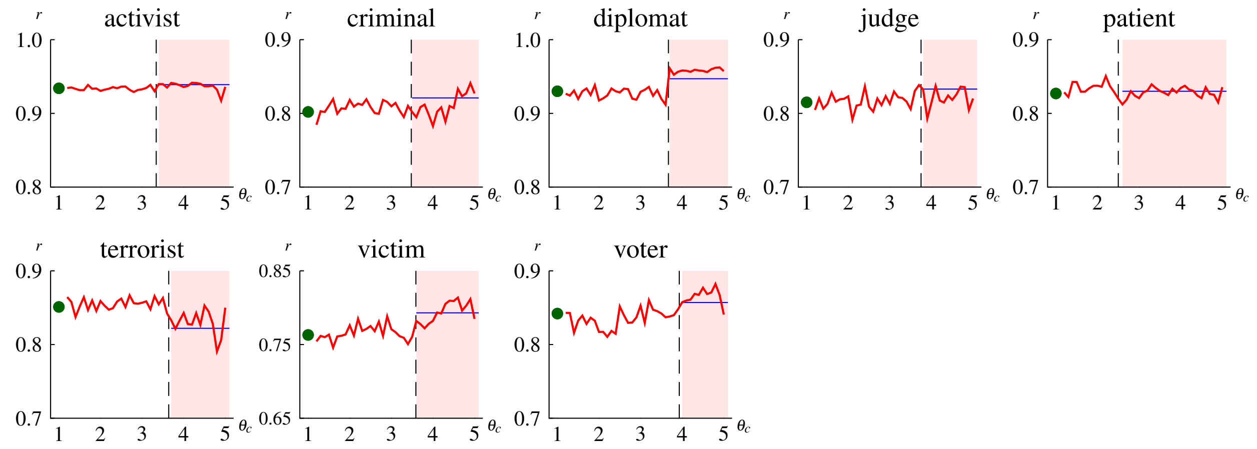

- Locative action

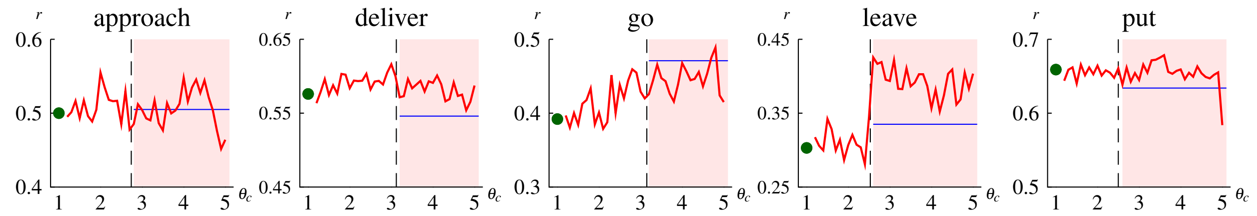

- Mental action

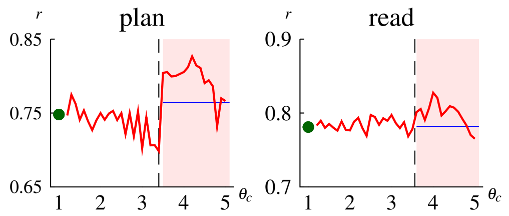

- Mental state

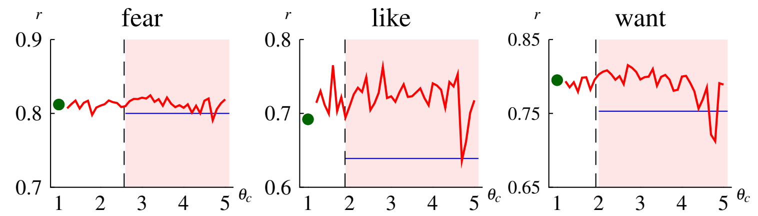

- Miscellaneous action

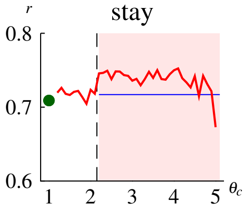

- Multimodal property

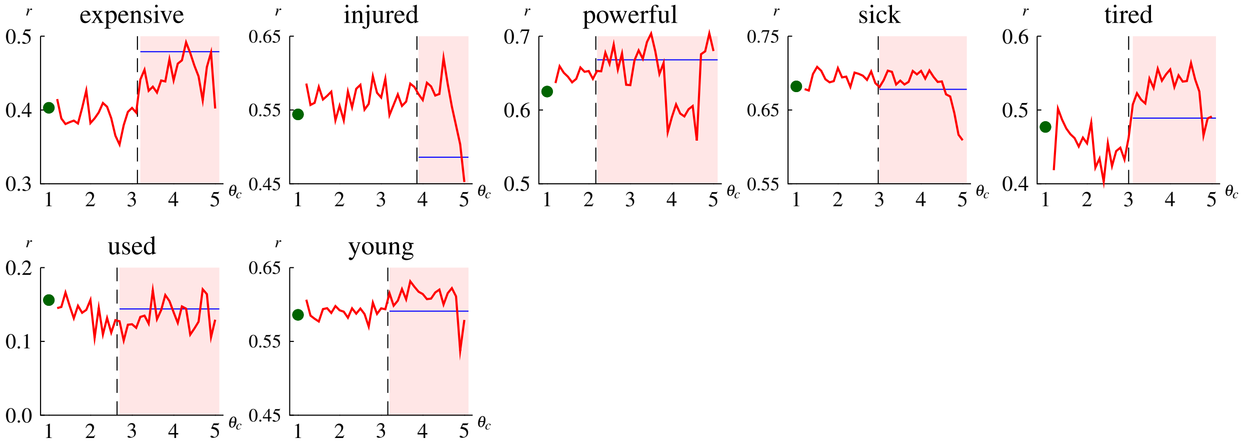

- Perceptual action

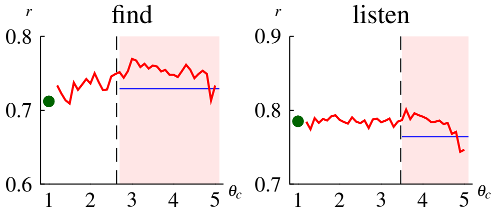

- Perceptual state

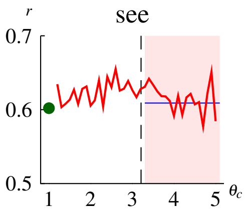

- Physical action

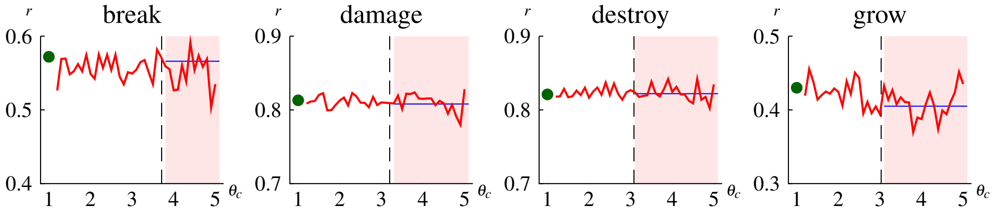

- Place

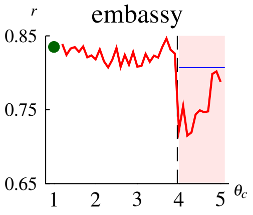

- Social action

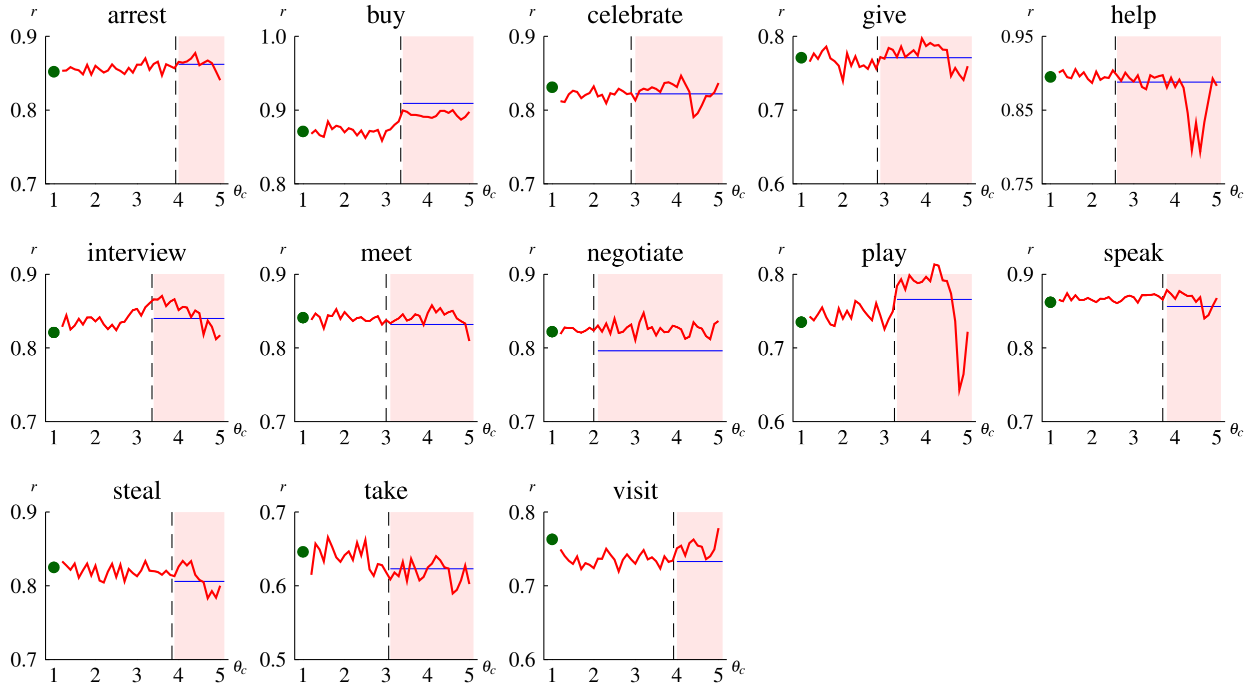

- Social construct

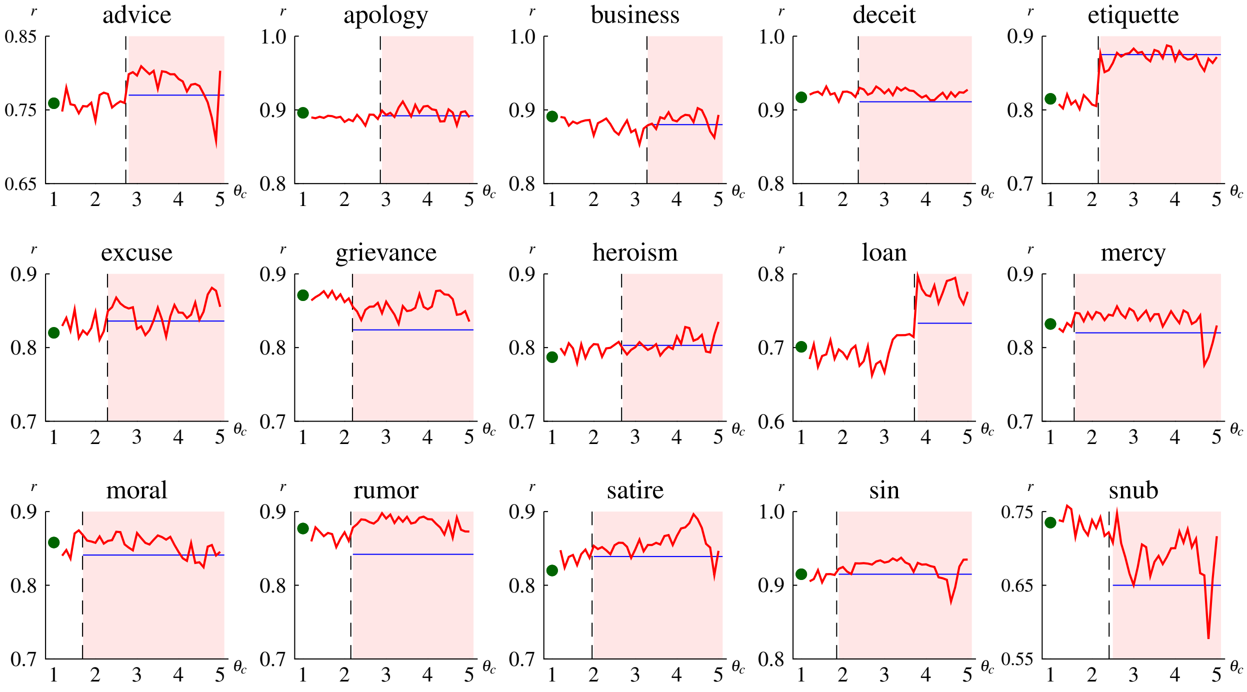

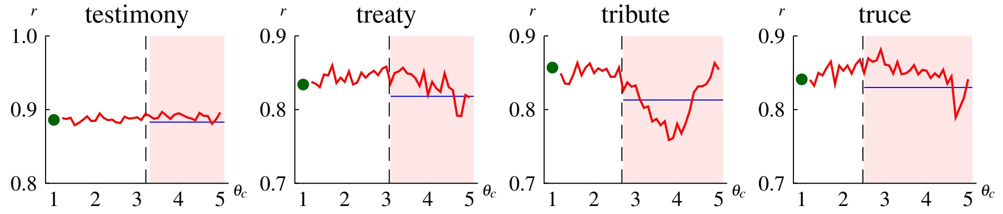

- Social event

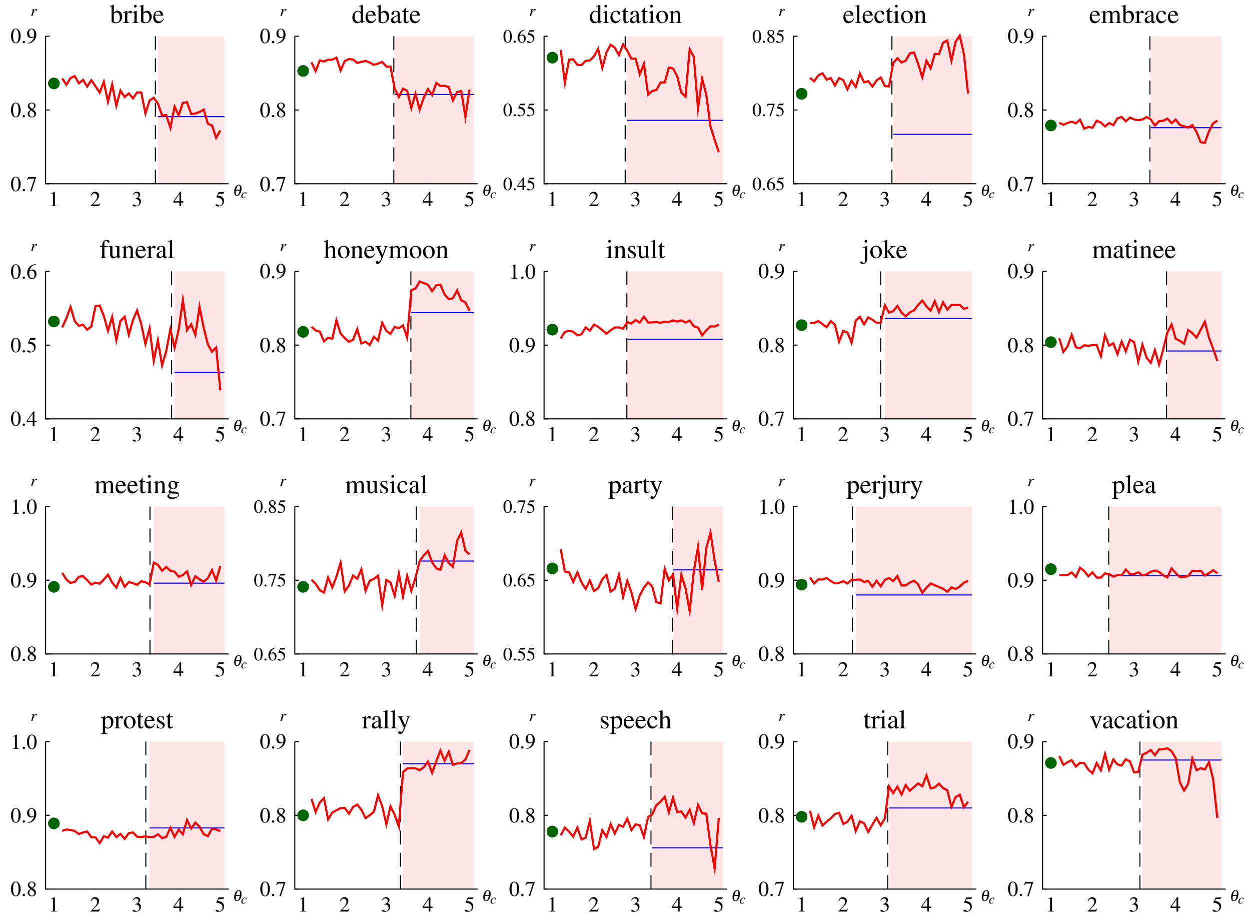

- Social property

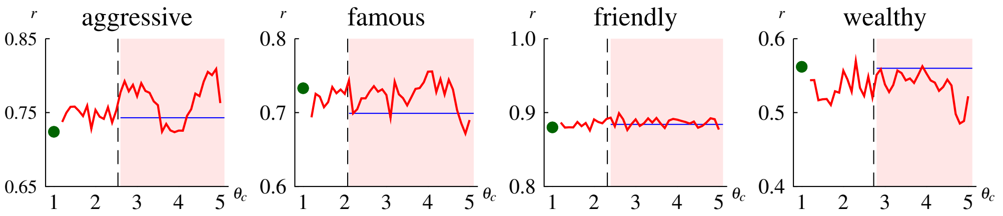

- Somatosensory property

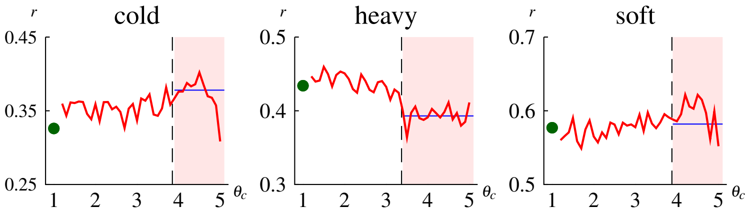

- Sound

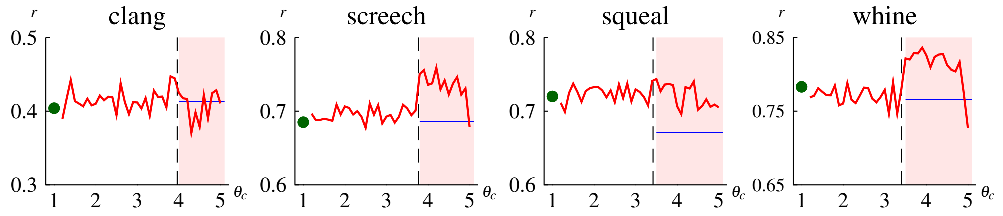

- Spatial property

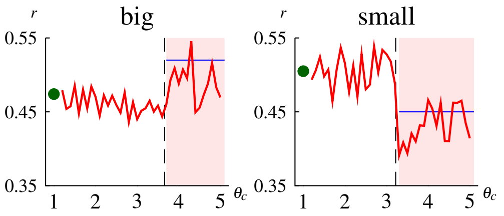

- Temporal property

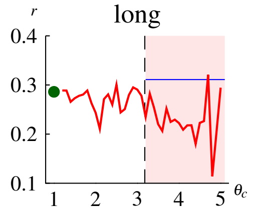

- Time period

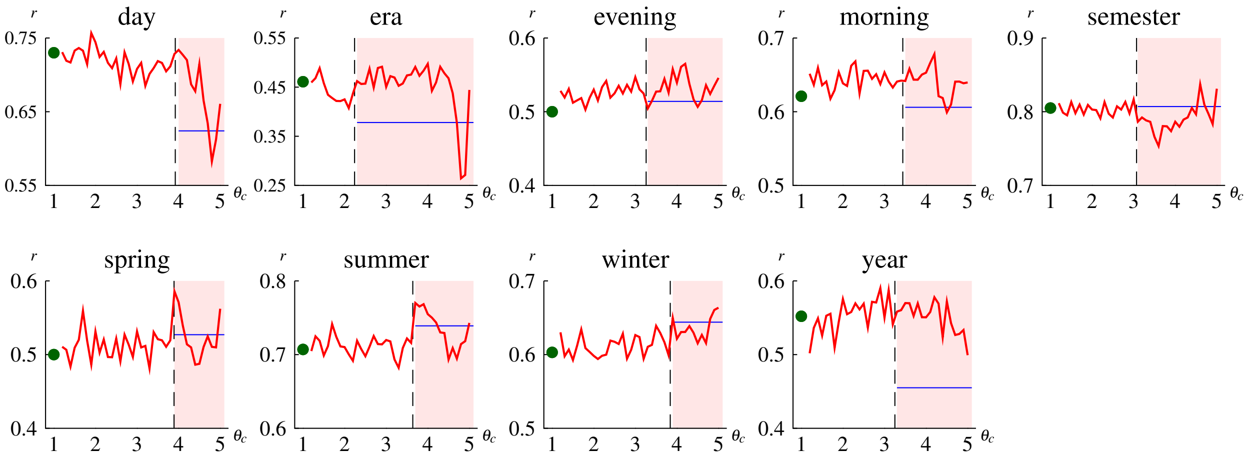

- Visual property

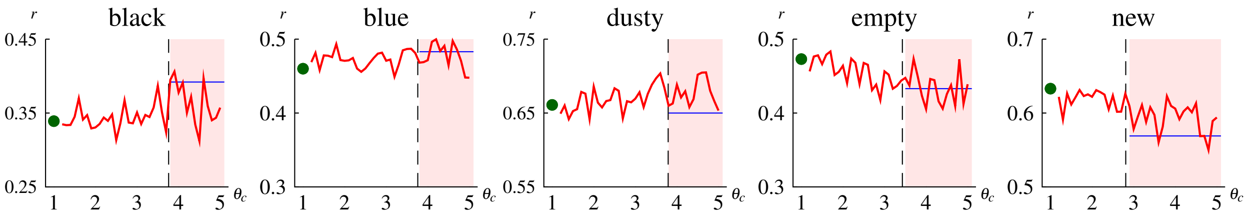

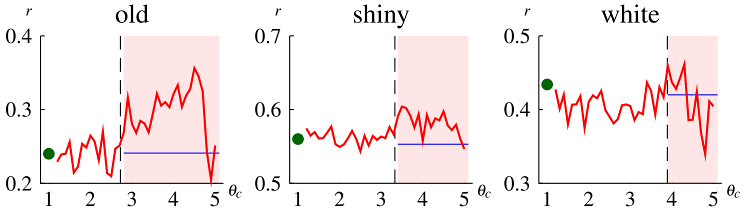

- Weather event

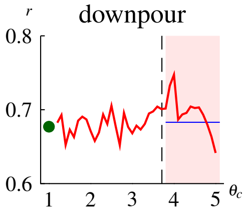

References

Binder, J. R., Conant, L. L., Humphries, C. J., Fernandino, L., Simons, S. B., Aguilar, M., & Desai, R. H. (2016). Toward a brain-based componential semantic representation. *Cognitive Neuropsychology*, *33*(3–4), 130–174. doi: 10.1080/02643294.2016.1147426
